# Bone mineral: A trojan horse for bone cancers. Efficient mitochondria targeted delivery and tumor eradication with nano hydroxyapatite containing doxorubicin

**DOI:** 10.1016/j.mtbio.2022.100227

**Published:** 2022-02-26

**Authors:** Yang Liu, Aftab Nadeem, Sujeesh Sebastian, Martin A. Olsson, Sun N. Wai, Emelie Styring, Jacob Engellau, Hanna Isaksson, Magnus Tägil, Lars Lidgren, Deepak Bushan Raina

**Affiliations:** a Department of Clinical Sciences Lund, Orthopedics, The Faculty of Medicine, Lund University, Lund, Sweden; b Department of Molecular Biology and the Laboratory for Molecular Infection Medicine, Sweden (MIMS), Umeå Centre for Microbial Research (UCMR), Umeå University, Umeå, Sweden; c Department of Theoretical Chemistry, Chemical Centre, Lund University, Lund, Sweden; d Medical Radiation Physics, Lund University, Lund, Sweden; e Department of Hematology, Oncology and Radiation Physics, Skåne University Hospital, Lund, Sweden; f Department of Biomedical Engineering, Lund University, Lund, Sweden

**Keywords:** Drug delivery, Nano and micro hydroxyapatite, Doxorubicin, Solid tumor, Osteosarcoma

## Abstract

Efficient systemic pharmacological treatment of solid tumors is hampered by inadequate tumor concentration of cytostatics necessitating development of smart local drug delivery systems. To overcome this, we demonstrate that doxorubicin (DOX), a cornerstone drug used for osteosarcoma treatment, shows reversible accretion to hydroxyapatite (HA) of both nano (nHA) and micro (mHA) size. nHA particles functionalized with DOX get engulfed in the lysosome of osteosarcoma cells where the acidic microenvironment causes a disruption of the binding between DOX and HA. The released DOX then accumulates in the mitochondria causing cell starvation, reduced migration and apoptosis. The HA+DOX delivery system was also tested in-vivo on osteosarcoma bearing mice. Locally delivered DOX via the HA particles had a stronger tumor eradication effect compared to the controls as seen by PET-CT and immunohistochemical staining of proliferation and apoptosis markers. These results indicate that in addition to systemic chemotherapy, an adjuvant nHA could be used as a carrier for intracellular delivery of DOX for prevention of tumor recurrence after surgical resection in an osteosarcoma. Furthermore, we demonstrate that nHA particles are pivotal in this approach but a combination of nHA with mHA could increase the safety associated with particulate nanomaterials while maintaining similar therapeutic potential.

## Graphical abstract

**Figure:**
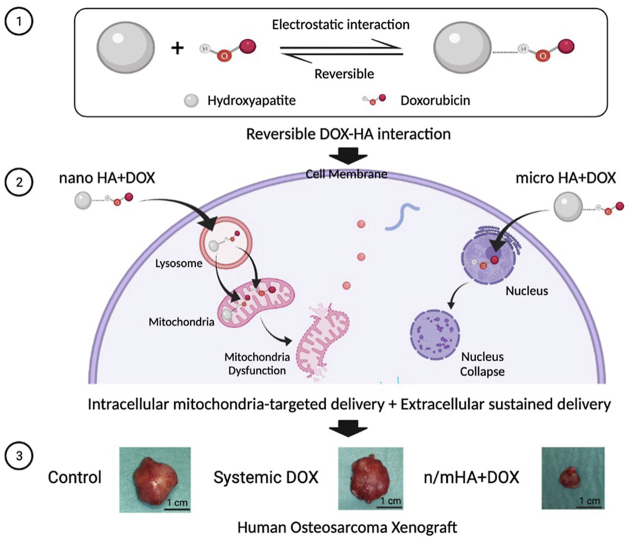

## 1 Introduction

Osteosarcomas are highly malignant solid tumors, usually affecting children and adolescents [1]. Methotrexate, doxorubicin (Adriamycin), and cisplatin (MAP) have been used as the backbone of standard treatment protocol and this drug regimen has significantly improved the prognosis for the affected individuals. However, for non-responders, i.e. the individuals that respond poorly, the options are few. Studies have shown that approximately 40% of osteosarcoma patients exhibit a poor response to the current chemotherapy protocol, with inferior 5-year survival of 45%–55% [2] and intensifying the treatment regimen by adding more drugs does not improve outcome but increases toxicity [3,4]. In order to improve outcome and alleviate side effects, there is an unmet need of efficiently delivering cytostatics to the tumor tissue in a targeted manner.

Hydroxyapatite (HA) is the main constituent of teeth and bones [5] and an elementary calcium phosphate with the chemical formula Ca_10_(PO_4_)_6_(OH)_2_, which has been extensively studied for various clinical applications [6]. HA can be synthesized and grinded into micro and nano sized particles. Nanoparticles in a defined volume have the advantage of having relatively larger surface area compared to microparticles, which dramatically improves the loading characteristics of an agent and consequently an increase in the drug delivery capacity (4–7). Recent research focus has been to biologically activate HA at the target tissue to increase drug efficacy, especially in bone cancer [7].

Previous studies have shown the anti-tumor effects of nHA on the metabolic viability of several types of cancer cells in-vitro [[8], [9], [10]]. However, the anti-tumor effect of pristine nHA seems to be insufficient to eradicate an established tumor, and therefore other methods need to be explored to enhance its cytotoxicity [[11], [12], [13], [14]]. Previous studies have shown that by adding chitosan or bovine serum albumin (BSA) as an intermediator, anti-tumor drugs can be incorporated on the surface of nHA for local delivery [15,16]. But the burst drug release due to rapid degradation of the coating materials compromise the efficacy. Instead of using an intermediary coating, chemical bonding as an alternative for drug loading to achieve a sustained and controlled drug release has been used in polymer-based nanomaterials [17]. However, it has rarely been explored on HA due to the difficulty of modifying the surface by adding reactive functional groups [18]. HA, as the hydroxyl endmember of the complex apatite group in a hexagonal crystal system, has fundamental adsorption capacity due to its positively charged surface (Ca^2+^) attracting anion pairing interactions with deprotonated carboxyl groups (–CO_2_^-^), and negatively charged groups, PO_4_^3−^, which promote interactions with protonated amines (–NH3^+^) [19]. It has been shown that various growth factors can be loaded on HA surface due to its physicochemical structure without impairing its bioactivity [20,21]. Furthermore, this molecule-HA interaction has also been verified in-vivo in bone regeneration and infections [7,22]. Pre-implanted HA can recruit circulating drugs with high affinity for HA, which eventually bioactivates the material and increases its therapeutic potential. Thus, drug loading and controlled delivery by taking the advantage of the chemical structure of HA might be an alternative to improve its tumor killing effect and facilitate its clinical translation.

The clinical translation of nanomaterials like nHA has been a concern due to the risk of particle migration from the implanted site. Leakage of the inorganic nanomaterials into the circulation might damage other vital organs [23]. Unlike nHA, HA microparticles are not able to enter the cells and have primarily been used for local delivery of various growth factors and drugs in bone due to its biocompatibility, and the ability to remain at the local site for years [24]. Furthermore, mHA based biomaterials have been used as bone substitute material for bone defect filling after trauma, infection and benign bone tumors [25]. Thus, combining mHA as a carrier with nHA for delivery might improve the safety with using nanomaterials [26].

Based on the physicochemical structure of HA, we hypothesized that cytostatics like DOX containing several accessible hydroxyl groups could be accreted on the surface of HA particles allowing for controlled local delivery of an anti-tumor drug with less off-target side effects. In addition, recent studies have indicated that particulate HA implanted in the body can attract systemically circulating drugs [7,22]. We therefore also hypothesize that systemically circulating DOX can be recruited by pre-implanted HA particles due to DOX-HA interaction. Thus, in this study, we aimed to explore the mechanism of accretion of a cytostatic like DOX, containing hydroxyl groups, to different size of HA particles both in-vitro and in-vivo. The efficacy of HA+DOX composite was tested on an aggressive human osteosarcoma cell line in-vitro and the mechanism of how the composite delivers the cargo was elaborately studied. Finally, a human osteosarcoma model was created in nude mice and the efficacy of HA+DOX composite on tumor suppression was evaluated.

## 2 Materials and methods

### 2.1 Study design

This study comprises of 3 main parts. Part 1: Validation of DOX-HA binding; Part 2: Intracellular pH-dependent lysosomal release and mitochondria-targeted delivery of DOX by nHA and Part 3: The efficacy of HA particles delivering DOX locally in an osteosarcoma xenograft. In part 1, DOX accretion to HA was explored by mixing DOX and HA for 24 or 48 ​h. Different dose of HA particles or DOX were set to detect the binding kinetics. Molecular dynamics was used to explore the mechanism of DOX-HA interaction. To detect in-vivo binding, rats with pre-implanted HA particles in the muscle pouch were given systemic injection of DOX. 24 h post-injection, samples were collected and scanned by IVIS Spectrum CT. In part 2, endocytosis of nHA+DOX was confirmed by flow cytometry. Then the cellular biodistribution of nHA+DOX was monitored by live cell confocal microscopy and further verified by TEM. The pH-dependent release of DOX was measured at acidic and physiological pH. The biological effect of nHA+DOX was detected on ATP synthesis, cell migration and viability. Finally, in part 3, the efficacy of HA particles delivering DOX locally was explored in an osteosarcoma xenograft model in nude mice. Tumor volume and weight were measured for tumor progression. PET/CT was used to detect the metabolically active tumor. Histological evaluation (H&E, Ki 67 and TUNEL) was used to further detect the microstructural changes within the tumor after intervention.

### 2.2 Materials

Doxorubicin was purchased from Merck (Doxorubicin, Hydrochloride - CAS 25316-40-9-Calbiochem, Merck, Germany). Hydroxyapatite powder was purchased from FLUIDINOVA, Portugal (micro hydroxyapatite powder, 10 ​μm and nano hydroxyapatite paste, <50 ​nm particle size). RPMI 1640 Medium and GlutaMAX™ Supplement were purchased from Thermo scientific, U.S.A. Heat inactivated fetal bovine serum (FBS) and MTT reagent was purchased from Sigma Aldrich, Germany. All the chemicals used in immunohistochemistry staining were purchased from Abcam: Citrate Buffer pH 6.0 (ab93678), Hydrogen Peroxide Blocking Reagent (ab64216), Protein Block (ab64226), Recombinant Anti-Ki67 antibody (ab16667), Antibody Diluent (ab64211), Goat Anti-Rabbit IgG H&L (HRP) (ab205718), DAB Substrate Kit (ab64238) and Hematoxylin Solution (Mayer's, Modified) (ab220365). TUNEL Assay Kit (ab206386) was also purchased from Abcam. ATPlite Luminescence Assay (6016943) was bought from PerkinElmer. Endocytosis inhibitors (Amiloride, LY2940002, MβCD, Dynasore) were purchased from Thermo Fisher. Hochst 33342, GFP-LAMP1, Mitotracker and Tom20 were all purchased from Thermo Fisher. 143B human osteosarcoma cells were purchased from American Type Culture Collection (ATCC). Athymic nude mice (Fox1^nu/nu^) were procured from Janvier Labs (France).

### 2.3 Physicochemical characterization of HA

Scanning electron microscopy (SEM) (Jeol JSM-7800F, Sweden) was used to analyze and compare the size distribution and surface of HA particles. The samples were dispersed on a SEM stub containing a double-sided carbon tape and imaged after sputter coating. The SEM instrument was operated at an operating voltage of 3.0 ​kV.

Transmission electron microscopy (TEM) was used to characterize the size and shape of the nHA and mHA used in this study. The nHA and mHA particles were dispersed in ethanol and a drop was put on a Cu/C grid. The ethanol was evaporated before evaluation under TEM (JEOL 3000F).

X-ray diffraction analysis (XRD) was used to further verify the presence of crystalline HA and its purity for both nHA and mHA and a detailed protocol is described elsewhere [27].

### 2.4 DOX binding to HA in-vitro

To evaluate the binding of DOX to HA in-vitro, 10 ​μg DOX dissolved in 1 ​mL phosphate-buffered saline (PBS) was added to 2 ​mL Eppendorf tubes containing varying amounts of nHA (10, 20, 40 and 80 ​mg). All the tubes were then left on a shaker to mix at 200 ​rpm for 24 ​h or 48 ​h. After the reaction, the particles were centrifuged and the supernatants collected. The particles were then thoroughly washed by resuspending them in 1 ​mL PBS, 5 times. The amount of DOX in the supernatants and the washed fractions was detected using a spectro-fluorimeter (Excitation-485 nm, Emission-580 nm).

To explore the relationship between the binding rate and the amount of DOX, 40 ​mg nHA was used for a 48 ​h reaction period and the dose of DOX was varied (10, 50, 100, 200, 400 and 800 ​μg).

To compare any differences in the binding affinity of DOX to nHA and micro sized HA (micro-HA), the same volume of both nHA and mHA were measured in an Eppendorf tube. 10 ​μg DOX dissolved in 1 ​mL PBS were mixed for 48 ​h and the binding % was detected as described above.

To explore the effect of proteins on DOX-HA binding, nHA particles (40 ​mg) were exposed to fetal bovine serum (FBS) for 1 ​h following which the particles were washed 3 times with PBS and then reacted with DOX solution (100 ​μg/mL). As a control, nHA particles were exposed to PBS for 1 ​h.

### 2.5 Molecular dynamics and binding free-energy estimates

Molecular dynamics (MD) simulations were performed with NAMD 2.14 in the NPT ensemble with periodic boundary conditions and binding free-energies were calculated using multi-state Bennett acceptance ratio. The detailed methodology used for the molecular dynamics studies are provided in the supplementary information.

### 2.6 DOX binds to pre-implanted nano or micro-sized HA in-vivo

A well-established abdominal muscle pouch model [28] was used to evaluate if systemically administered DOX seeks ectopically implanted micro and nano HA particles in-vivo. 25 ​mg nHA and mHA particles were implanted bilaterally into two muscle pouches on either side of the abdominal midline in male, Sprague-Dawley rats (n ​= ​20) under anesthesia. A collagen sponge (4 ​mm diameter and 2 ​mm height) was also implanted to confirm that the binding is specific to HA. Another pouch was created in the muscle by making a blunt cut and was used as negative control. Thus, a total of 4 pouches were created containing different materials (nHA, mHA, collagen sponge, empty muscle pouch) and each implant was separated from the other by a minimum distance of 2 ​cm. At day 7 after operation/post-implantation, a systemic DOX injection (0, 0.5, 1.0 and 2.0 ​mg) was given subcutaneously to 5 rats for each dose. 24 h after the injection, the rats were euthanized by CO_2_ asphyxiation and the samples in the muscle pouch were collected and scanned on an IVIS spectrum in-vivo imaging system (PerkinElmer, Waltham, Massachusetts, USA). Epi-fluorescent measurements were obtained at excitation 487 ​nm and emission 580 ​nm. When comparing the binding rate between nHA and micro-HA, the fluorescence was also normalized to the BET surface area of the particles provided by the manufacturer.

To further verify that the IVIS signal indeed represented the implanted materials, hematoxylin and eosin (H&E) and Alizarin Red S staining was performed to check the materials and the microstructure of the tissue in the samples. Samples from each group were fixed in 4% (v/v) formalin solution for 24 ​h after which they were processed using standard paraffin embedding procedures and cut to a thickness of 5 ​μm for staining (HM355S, Thermo Fisher Scientific, MA, USA).

### 2.7 Intracellular uptake of nHA loaded with DOX and its intracellular distribution

For cell experiments, nHA+DOX was prepared by mixing 40 ​mg nHA with 1 ​mL DOX solution (1 ​mg/mL) for 48 ​h. After mixing, the DOX in the supernatant and washed fractions were measured. To make the stock solution for cell experiments, 40 ​mg nHA containing 920 ​μg DOX was used to make 1 ​mg/mL nHA+DOX stock (23 ​μg/mL DOX). Different dilutions were used to prepare cell culture medium for various experiments.

143B human osteosarcoma cells were cultured in RPMI 1640 supplemented with 10% FBS, 1% penicillin, 1% streptomycin and 1% Glutamax. The media was replaced every 2–3 days.

For live cell confocal microscopy, 143B cells were treated with increasing concentration of nHA+DOX for 24 ​h in an incubator at 37 ​°C. The extracellular nHA+DOX was washed with complete media three times, followed by counterstaining the cells with Hoechst 33342 (2 ​μM) for 10 ​min at 37 ​°C. Subsequently, the cells were washed with complete media and visualized using Leica SP8 inverted confocal system (Leica Microsystems) equipped with a HC PL APO 63x/1.40 oil immersion lens. Images were captured and processed using the LasX software (Leica Microsystems).

For 143B fixed cell immunofluorescence, cells were grown on coverslip-bottom 8-well chamber slide (μ-Slide, ibidi) and fixed in 2% paraformaldehyde for 30 ​min at room temperature (RT) and subsequently permeabilized in 0.25% Triton X-100 (15 ​min). Cells were blocked with 5% fetal bovine serum (60 ​min, RT) followed by incubation with primary antibodies against Tom20 (#612278, BD Biosciences) at room temperature. Cells were then washed with PBS (3 times) and subsequently incubated with Alexa555-conjugated secondary antibodies (60 ​min) at RT. Cells were counterstained with Hoechst 33342 (2 ​μM) for 5 ​min.

For co-localization experiments, 143B cells were transfected with lysosomal marker, GFP-LAMP1 (Invitrogen) followed by treatment with nHA+DOX or nHA (50 ​μg/mL) for 4 ​h. Cells were washed in complete media and visualized using Leica SP8 inverted confocal system (Leica Microsystems). Fluorescence intensity profiles were generated using the plot profile command in ImageJ. Confocal microscopy images were processed using ImageJ–FIJI distribution [29].

For cellular uptake of nHA+DOX (50 ​μg/mL, 24 ​h), 143B cells were treated in the presence or absence of a given pharmacological inhibitor as indicated in the corresponding figure legend. At the end of treatment, cells were detached with trypsin and mixed with trypan blue to quench any traces of extracellular nHA+DOX. Cells were analyzed by flow cytometry (BD Accuri). Live cells were gated and cellular uptake of nHA+DOX was represented as mean fluorescence intensity (MFI). For inhibitor experiments, data was normalized to the nHA+DOX treated cells and expressed as a percentage. For concentration dependent increase in cellular uptake of nHA+DOX, data was expressed as mean fluorescence intensity (MFI).

To further confirm the nanoparticles localization in the cells using TEM, 7×10^5^ 143B cells were seeded on T25 culture flasks 24 ​h before intervention. Cells were either untreated or treated with nHA+DOX (250 μg/flask in 5 ​mL medium, containing 5.625 ​μg DOX) for a culture period of 4 ​h. The cells were then detached by Trypsin-EDTA (0.25%) and centrifuged to form a cell pellet (400 ​g, 4 ​min). Cell pellets were fixed with 4% formaldehyde for 12 ​min, followed by 3% glutaraldehyde solution in 0.1 ​M phosphate buffer (pH ​= ​7.4) for 2 ​h, post-fixed in osmium tetroxide (3%) for 2 ​h, dehydrated in graded acetone and embedded in Araldite (Fluka, Buchs, Switzerland). Ultrathin sections (100 ​nm) were collected on 200 mesh copper grids and contrasted using both lead citrate and uranyl acetate and then examined with a transmission electron microscope (JEOL 3000F). To explore the cellular uptake of mHA particles, 7×10^5^ 143B cells were seeded on T25 culture flasks 24 ​h before intervention. Cells were treated with mHA particles (100 ​μg/mL) for 24 ​h and then processed for TEM as described above.

To explore the pH dependent release pattern for nHA+DOX, 40 ​mg nHA was reacted with 100 ​μg/mL DOX solution for 48 ​h. The nHA+DOX composite was then placed in PBS at various pH (2.5, 5 and 7.4). The supernatants were collected at each time point (1, 6, 12, 24 and 72 ​h) and replaced with another 1 ​mL PBS with relevant pH. The DOX in the supernatants were measured and calculated for drug release. For mHA+DOX, 100 ​mg mHA was used to react with 1 ​mL DOX solution (100 ​μg/mL). The release was determined as described above.

### 2.8 nHA delivers DOX to mitochondria and its effect on human osteosarcoma cells

To track the movement of nHA+DOX inside the cells after being taken up, live cell confocal microscopy was used to monitor the particles up to 24 ​h. The procedures are described as above (section 2.6). For co-localization of Mitotracker and nHA+DOX, 143B cells were treated with nHA+DOX or nHA (50 ​μg/mL) for 4 ​h. Subsequently, cells were counterstained for Mitotracker (500 ​nM) and Hoechst 33342 for 30 ​min. Cells were washed in complete media and visualized using Leica SP8 inverted confocal system (Leica Microsystems).

For TEM, samples were processed as previously described (section 2.7). Cells were fixed 24 ​h after being given nHA+DOX treatment.

Luminescent ATP detection assay kit (ab113849: Abcam, Cambridge, MA, USA) was used to measure ATP produced by human osteosarcoma cells treated with various interventions. In brief, cultured 143B cells and ATP standard dilution series were prepared. D-Luciferin and firefly luciferase reagents were added to the reaction mix, stirred and incubated for 10 ​min. Luminescence from luciferase activity was recorded using a spectro-fluorimeter. A standard curve was plotted and luminescent units from each sample were interpolated to calculate the absolute ATP concentration.

For cell migration, 143B cells were seeded at a density of 1×10^5^ ​cells/well in 48-well culture plates. 2 days after cell seeding, A wound was artificially created by scratching the cell monolayer with a 1 ​mL pipette tip in the middle of the well. Plates were washed with warm PBS to remove the detached cells and then different interventions were added. Normal cell culture medium and medium containing nHA were used as negative controls. Medium containing DOX (2.3 ​μg/mL) was used as positive control. Medium containing nHA+DOX (25–100 ​μg/mL, equal to 0.575–2.3 ​μg/mL DOX) were used as treatment groups. Wound closure was compared at t ​= ​0 and t ​= ​24 ​h, and the images were taken on an inverted microscope. The wound closure % in each group was calculated according to the following equation: Wound Closure % = ((Wound Area_0h_-Wound Area_24h_)/Wound Area_0h_)x100.

### 2.9 Cytotoxicity of nHA+DOX on human osteosarcoma cells and the biocompatibility of HA particles with osteoblasts

143B (1×10^4^ ​cells/well) were seeded on 96-well plates. Complete cell culture medium as described above containing nHA (25 ​μg/mL) or nHA-DOX (25–100 ​μg/mL, containing 0.575–2.3 ​μg/mL) were given to cells 24 ​h after seeding and incubated for 1 day. Medium containing DOX (2.3 ​μg/mL) was used as positive control. A detailed protocol is described before [27].

To explore the biocompatibility of HA particles, various concentrations of HA particles were given to the 143B cells and MC3T3 cells. Medium without any HA particles was taken as control. For 143B cells, the concentrations of 12.5, 25 and 50 ​μg/mL were given to cells for 24 ​h. For MC3T3 cells, the concentrations of 50, 100 and 200 ​μg/mL were used for 48 ​h.

### 2.10 Tumor eradication effects of DOX loaded HA in highly aggressive human osteosarcoma model after resection

For human osteosarcoma xenograft model, subcutaneous cell injection of human osteosarcoma cells (2×10^6^ human 143B osteosarcoma cells) was performed. On day 12, the core of the established tumors (average volume: 72 ​± ​27 ​mm^3^) was resected by a 2 ​mm biopsy punch to mimic post-surgery chemotherapy. 43 mice were randomly assigned to 5 groups: Control group (G1) (n ​= ​8), DOX IV group (G2) (n ​= ​9), nHA+DOX (G3) (n ​= ​8), mHA+DOX group (G4) (n ​= ​9) and n/mHA+DOX group (G5) (n ​= ​9). All the particle pellets used in this experiment were casted in a mold with 3 ​mm diameter using 50 ​μL hyaluronic acid (10 ​mg/mL) to keep the shape of the pellets for implantation. For G2, 40 ​μg DOX was given intravenously to each animal. In G3, each nHA pellet contained approximately 43 ​μg DOX. In G4, two mHA pellets containing a total of 40 ​μg DOX were implanted and for G5, one and half n/mHA pellets containing 46 ​μg DOX were implanted. Tumor volume was measured during the follow-up period twice/week.

### 2.11 PET-CT imaging for detection of metabolic activity in the tumors

A Nano PET/CT scanner (Mediso, Hungary) was used to measure the metabolic tumor activity, 20 days post intervention using ^18^F-Fluorodexoyglucose (^18^F-FDG) as a tumor metabolic activity marker. ^18^F-FDG (42.6 ​± ​4.2 MBq) saline was injected intraperitoneally in a 100–500 ​μL final volume in awake mice. At t ​= ​63 ​± ​3 ​min, whole-body PET images were acquired on a high-resolution (voxel size ​= ​300 ​μm) small animal PET/CT scanner as described previously [27]. The acquired PET images were reconstructed by the Tera-Tomo™ three-dimensional (3D) PET image reconstruction software (Mediso, Hungary) and an overlap CT-PET image was first used to confirm the location of the tumor and the uptake of ^18^F-FDG. Quantification of ^18^F-FDG in each tumor was performed using an established protocol described earlier [27].

### 2.12 Immunohistochemistry staining to detect cell proliferation and apoptosis within the tumor tissue

All the animals were sacrificed 25 days post treatment. The weight of the excised tumors was noted for all samples. Histology was used to compare the microstructure of the harvested tumor tissues. Samples from each group were fixed in 4% (v/v) formalin solution for 24 ​h. 4 ​mm biopsy punch was used to get uniform cylinders from each tumor sample. 3–4 cylinders were prepared to represent the histology of the whole tumor for the big tumor samples and 1–2 cylinders for the small tumor samples. The cylinders were dehydrated in increasing ethanol gradient, treated with xylene and embedded in paraffin. Paraffin blocks were sectioned using a semi-automatic microtome (HM355S, Thermo Fisher Scientific, MA, USA) to a thickness of 3 ​μm and stained with hematoxylin and eosin (H&E).

For immunohistochemistry staining, the samples were sectioned to 3 ​μm thickness and all the slides were placed in 37 ​°C for 48 ​h. The slides were then deparaffinized and rehydrated. Citrate buffer (pH ​= ​6.0) in distilled water was used for antigen retrieval for Ki 67. Hydrogen peroxide blocking reagent (8 ​min) was used to inactivate the endogenous peroxidase activity. Protein Block was then used for 30 ​min to block non-specific antibody binding sites. The slides were incubated with anti-Ki67 (1:200) antibody overnight in a humidified chamber at 4 ​°C. Subsequently, secondary antibody was applied at 1:1000 dilution for anti-Ki67 for 30 ​min at room temperature. The color developing DAB solution was added and incubated for 3 ​min. Lastly, hematoxylin was added to the slides for 1 ​min. The slides were then dehydrated, cleared in xylene and mounted using a xylene based mounting medium. For immunohistochemistry staining, only secondary antibody staining with the above steps were done to avoid false positive stains and confirm the reliability of antibody-based staining.

A commercially available In Situ Apoptosis Detection Kit (Abcam) was used to detect apoptosis in tumor samples from each group (n ​= ​5) according to the manufacturers protocol. Briefly, paraffin sections were deparaffinized in xylene and rehydrated in a graded alcohol series before the treatment with Proteinase K. Then 3% H_2_O_2_ was added to inactivate endogenous peroxidases. Apoptotic cells were labelled with TdT Enzyme, followed by incubation with conjugate. In the case of negative controls, TdT was substituted with distilled water instead of TdT Enzyme. The signal was detected using 3,3′-diaminobenzidine (DAB) substrate and sections counterstained with Methyl Green. To quantify TUNEL positive cells, 5 high magnification fields (HMF, 20×) were randomly chosen from each sample and positive cells were counted to represent cell apoptosis for each sample.

### 2.13 Statistical analysis

All data are presented as Mean ​± ​SD and checked for normality before statistical analysis. To evaluate DOX binding to HA, a student t-test was used to analyze in-vitro DOX binding (timing and size of HA) and in-vivo DOX binding (size of HA). Repeated Measures ANOVA and Dunn's multiple comparisons test was used to analyze the in-vivo DOX binding affinity comparing nHA, mHA and collagen to surrounding muscles. Paired *t*-test was used to compare DOX binding affinity to differently sized HA (nHA vs mHA) particles at each dose. For in-vitro cell experiments, one-way ANOVA with Tukey's multiple comparisons test was used for cell viability, cell migration and intracellular ATP level. For in-vivo xenograft model, Kruskal-Wallis test with Dunn's multiple comparisons test or One-way ANOVA with Dunnett's multiple comparisons test for comparing treatment groups (G2-G5) to control group (G1) was used to test the difference in tumor volume, tumor weight, total ^18^F-FDG uptake, the expression of Ki-67 and TUNEL within the tumor. The differences among the other 4 treatment groups (G2-G5) were further tested with Kruskal-Wallis and Dunn's multiple comparisons or One-way ANOVA with Tukey's multiple comparisons, based on the distribution of data. All statistical analysis was carried out in Prism8 (GraphPad PRISM 8.2.1, USA) and p ​< ​0.05 was considered statistically significant.

### 2.14 Animal ethics statement

All animal procedures were approved (Ethical approval number 5.8.18–15288/2019 and 5.8.18–01018/2020) and performed in accordance with the directives of the Swedish regulatory authority for the use of animals for experimental purposes (Jordbruksverket). Furthermore, we have also adhered to the ARRIVE guidelines for providing information pertaining to the animal experimentation.

## 3 Results

### 3.1 Validation of DOX-HA binding

#### 3.1.1 DOX binding to HA is dependent on reaction time and particle size

**Fig. 1:**
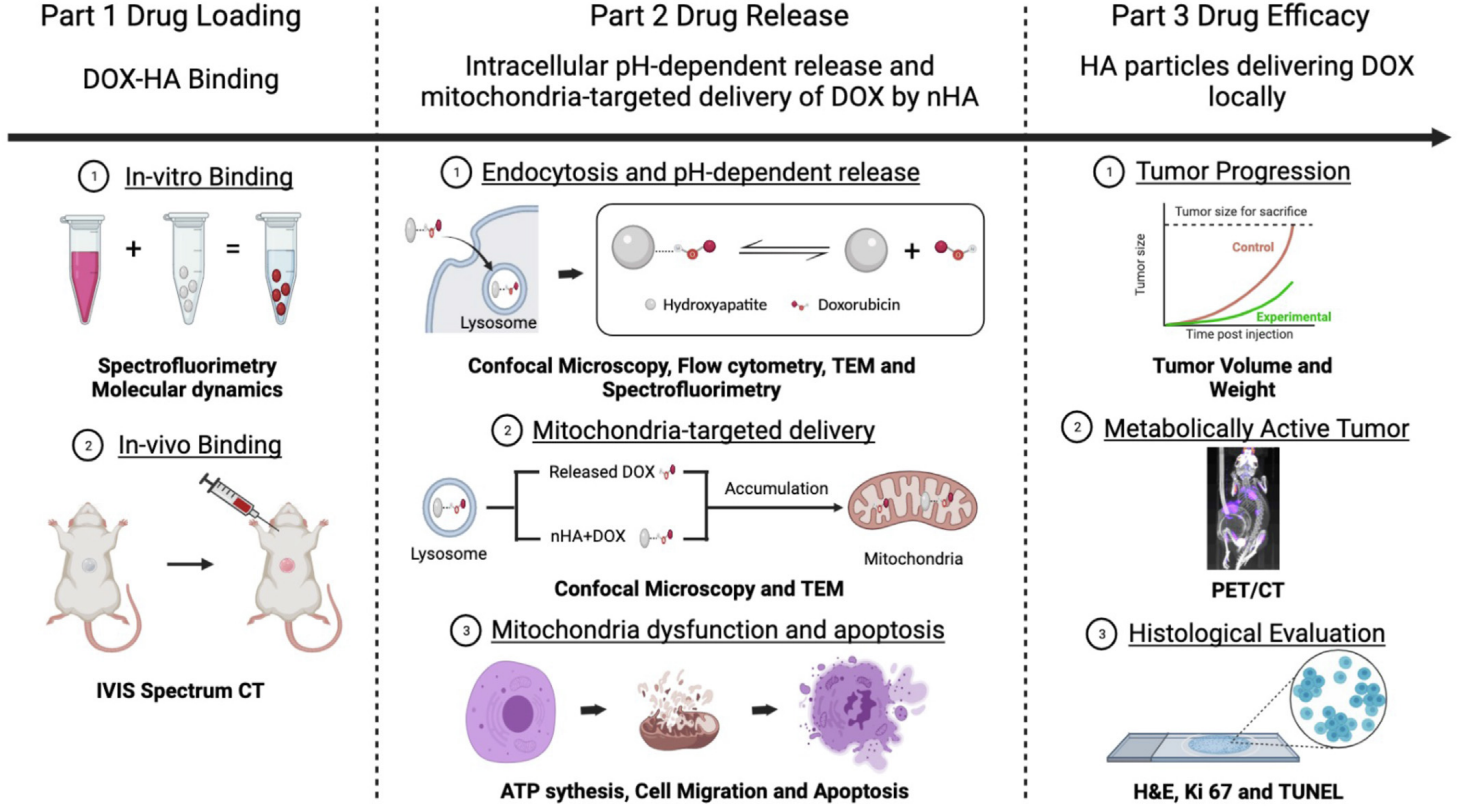
Schematic of the study design and the evaluation techniques used in the evaluation of DOX-HA binding, intracellular mitochondria-targeted delivery of DOX and the efficacy of DOX-HA complex in a relevant human osteosarcoma model. This figure was made on BioRender.com.

**Fig. 2:**
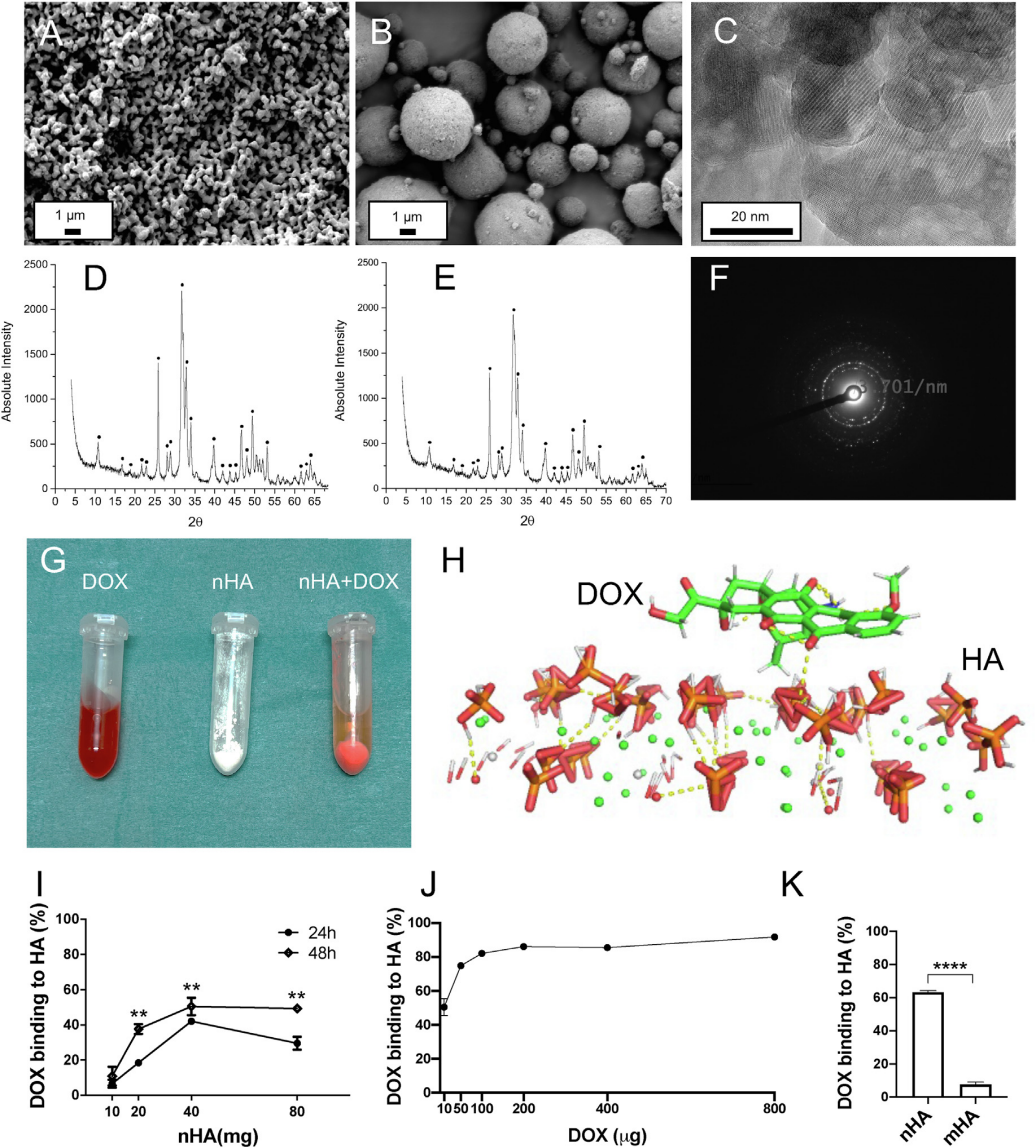
Physicochemical characterization of different sized HA and evaluation of HA-DOX interaction in-vitro. (A–B) SEM images showing shape and size distribution for nano (A) and micro (B) HA particles. (C,F) TEM images showing typical crystalline structure of nHA and its diffraction pattern. (D–E) XRD data confirming the presence and purity of nHA (D) and mHA (E). • indicates peaks for hydroxyapatite. (E) Has been modified from our earlier work [27] (G) DOX was reacted with increasing concentrations of nHA particles over a period of 24 or 48 ​h. Notice nHA particles turn pink while the solution of free DOX reduces in intensity after the DOX-HA reaction. (H) Equilibrated binding pose of DOX on the (001) plane of HA from molecular dynamics simulation. (I) Line graph shows binding kinetics of DOX to increasing concentration of nHA at two different time points, 24 ​h and 48 ​h. (J) Line graph shows binding kinetics of increasing concentration of DOX to 40 ​mg nHA at 48 ​h time point. (K) Comparison of DOX binding to nHA or mHA. A student t-test was used to analyze in-vitro DOX binding (24 ​h vs 48 ​h and nHA vs mHA). ∗∗ indicates p ​< ​0.01, ∗∗∗∗ indicates p ​< ​0.0001.

Scanning electron microscopic analysis was performed to examine the ultrastructure of HA particles. Rod shaped nHA particles were observed with a length span of 20–50 ​nm mHA particles were arranged as uniform spheres with a diameter of about 1–10 ​μm. (Fig. 1, Fig. 2A and B, Fig.S1). The purity of HA was further confirmed by XRD. Data analysis from XRD measurements showed that both nHA and mHA had characteristic crystalline hydroxyapatite peaks with 100% purity (Fig. 2D and E). TEM+EDX verified that nHA was clearly crystalline with HA specific diffraction pattern (Fig. 2C,F). The presence of calcium in HA nanoparticles was also verified (Fig.S2). DOX presented with an orange or red color when dissolved in PBS. After mixing DOX solution with pure white nHA, the red color in the liquid became lighter and white nHA turned pink and did not change even after 5 washes, which was seen as an indirect indication of DOX interaction with nHA (Fig. 2G). Molecular dynamics data indicated that DOX accretion to HA is mainly because of electrostatic interactions with calcium ions of HA (Fig. 2H). To further explore DOX-HA interaction, various potential factors including binding kinetics and increasing concentration of HA or DOX were investigated. For both time points, the binding rate showed an increasing trend up until the dose of 40 ​mg nHA after which the binding stayed relatively constant. The highest DOX binding of 42% was observed at 24 ​h and 50.5% at 48 ​h. Furthermore, the binding rate was significantly higher for 48 ​h compared to 24 ​h at all HA doses except 10 ​mg (Fig. 2I). To explore the relationship between the binding rate and DOX dose, 40 ​mg nHA was used to react with increasing doses of DOX (10, 50, 100, 200, 400 and 800 ​μg). The binding rate increased with increasing dose of DOX, with the highest binding rate being 91.8% (for 800 ​μg DOX) (Fig. 2J). Pre-treatment of HA with serum proteins did not affect the DOX binding significantly and the DOX-HA binding capacity reduced only by 7% when the HA particles were exposed to serum proteins for 1 ​h compared to pure nHA (Fig. S3). By normalizing the weight of the HA particles used in the experiment to the surface area (BET analysis performed by the manufacturer), nHA showed a substantially higher DOX binding of about 63.3% compared to mHA with 7.65% (Fig. 2K).

#### 3.1.2 Implanted HA particles recruit circulating DOX in-vivo

**Fig. 3:**
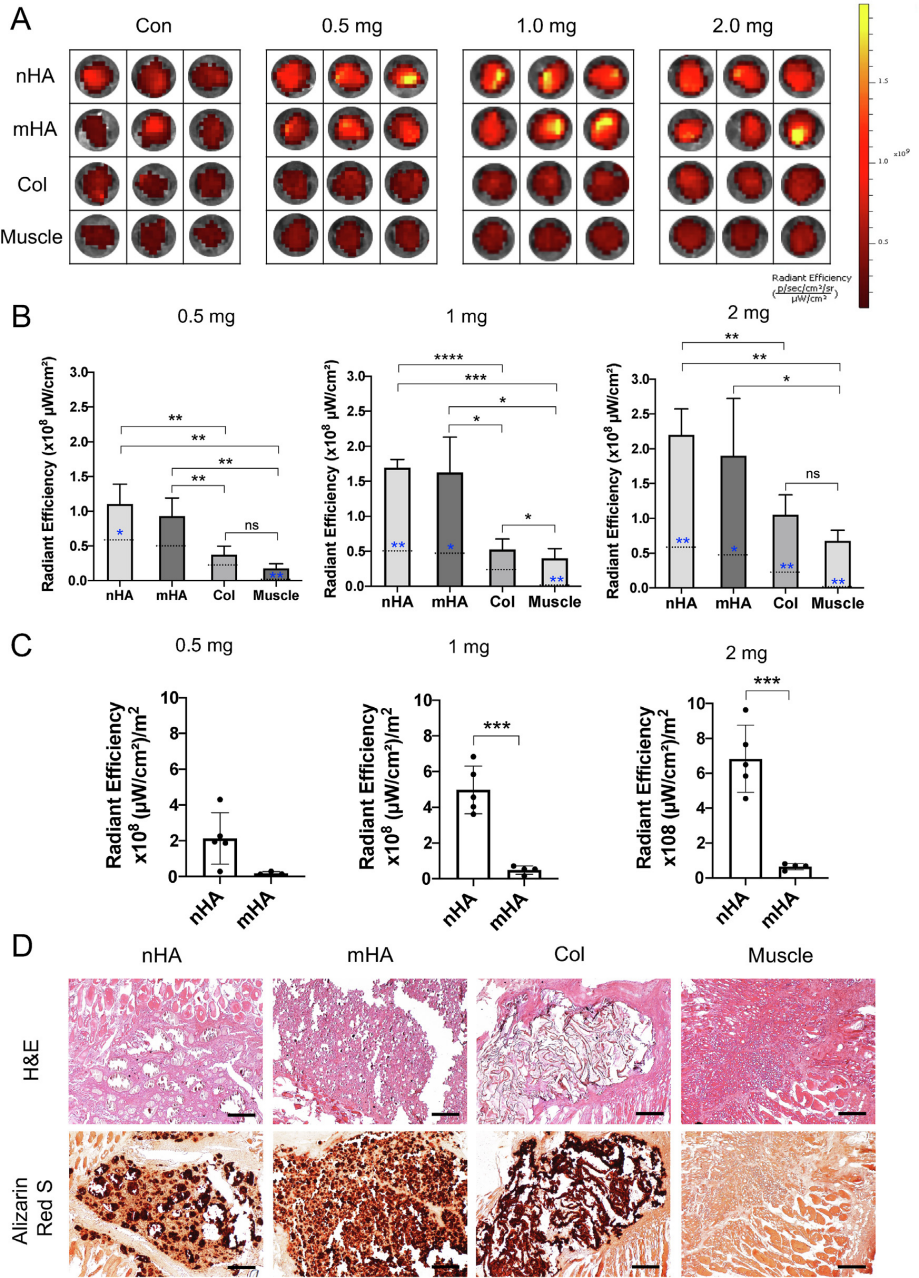
Systemically circulating DOX binds to pre-implanted nHA or mHA in-vivo. (A) Representative IVIS images showing the presence of DOX in different implanted materials. Notice an increased fluorescent signal in nHA and mHA compared to collagen sponge or surrounding muscle. (B) Implanted nHA and mHA could recruit more DOX compared to the collagen sponge, the surrounding muscle or the respective material without DOX injection. Dashed blue line on each bar represents the signal from respective materials without DOX. Repeated Measures ANOVA and Dunn's multiple comparisons test was used to analyze the in-vivo DOX binding affinity comparing nHA, mHA and collagen to surrounding muscles. (C) DOX has a stronger affinity to nHA compared to mHA and the affinity was dose dependent. Paired *t*-test was used to compare the two different HA materials (nHA vs mHA). (D) H&E verifies the presence of the implanted materials in the muscle pouch 7-days post implantation (D, top panel). Presence of HA was further confirmed by Alizarin Red S stain (D, bottom panel) with calcium being stained as red to black. The HA particles were aggregated together after implantation. Scale bar ​= ​100 ​μm ∗ indicates p ​< ​0.05, ∗∗ indicates p ​< ​0.01, ∗∗∗ indicates p ​< ​0.001 and ∗∗∗∗ indicates p ​< ​0.0001.

In-vitro data indicated a strong interaction between DOX and HA. To further confirm the binding affinity of DOX to HA, nHA and mHA were implanted in the abdominal muscle pouch of rats. A collagen sponge was also implanted as a control. Surrounding muscles were taken as negative control. When scanned with IVIS, samples from nHA or mHA group showed higher fluorescence signals than the collagen sponge and surrounding muscles (Fig. 3A, for all samples see Fig.S4). The fluorescence in the collagen sponge was similar to the surrounding muscle (Fig. 3A). DOX that was recruited by HA irrespective of particle sizes was 2–3 times more than surrounding muscle (Fig. 3B). The recruitment of the drug was positively related to the dose of DOX (Fig. S5). After normalizing the weight of particles to its surface area (binding sites), nHA showed a 10.3–11.5 times stronger binding affinity for DOX compared to mHA (Fig. 3C), corroborating with the in-vitro results. Histology showed that 1 week after implantation, nHA and mHA still remained present at the implantation site and that a fibrotic tissue capsule had formed around the particles. The collagen sponge had a typical net like structure in the muscle. Further confirmation by Alizarin Red S stain verified the presence of calcium in the HA (Fig. 3D).

### 3.2 Intracellular pH-dependent lysosomal release and mitochondria-targeted delivery of DOX by nHA

#### 3.2.1 Intracellular delivery of DOX by nHA. A pH-dependent drug release through lysosome

**Fig. 4:**
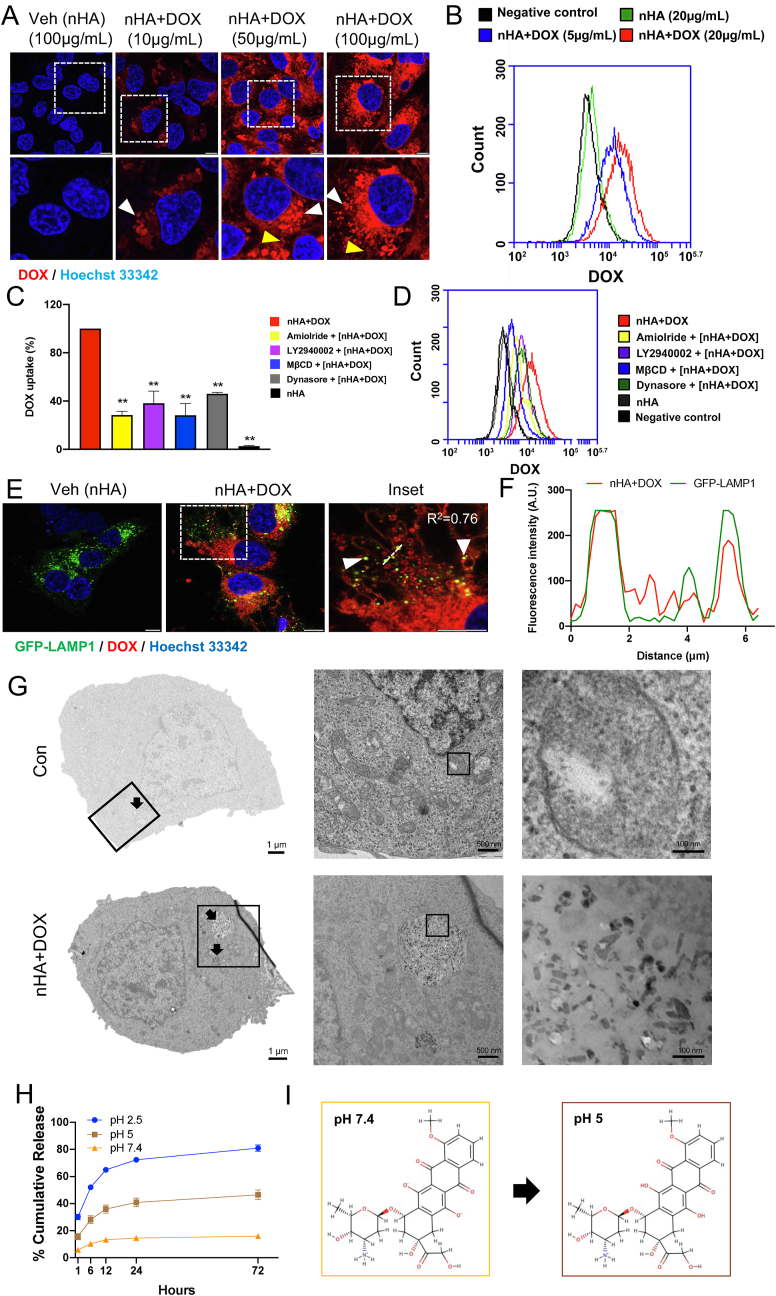
nHA+DOX is internalized by tumor cells via endocytosis and it accumulates in the lysosome (A) 143 ​B cells treated with increasing concentration of nHA+DOX for 24 ​h. Cellular distribution of DOX (red) was investigated by confocal microscopy. Nuclei were counterstained with Hoechst 33342 (blue). The arrowhead (white) indicates accumulation of nHA+DOX in cytoplasmic vesicles while the arrowhead (yellow) indicates localization of nHA+DOX in cytosolic filaments. Data is representative of two independent experiments. Scale bar indicates 10 ​μm. (B) 143B cells were treated with increasing concentration of nHA loaded with DOX for 24 ​h. Cellular uptake of DOX was assessed by flow cytometry analysis. Representative plot from two independent experiments is shown. (C–D) 143B cells were treated with nHA+DOX (20 ​μg/mL) for 24 ​h with or without endocytosis inhibitors; Amiloride (1 ​mM), methyl β-cyclodextrane (2.5 ​mM), LY294002 (20 ​μM) or Dynasore (50 ​μM). The inhibitors were added 30 ​min prior to the addition of nHA+DOX. Data is representative of three experiments; bar graphs show Mean ​± ​SD. Significance was determined from replicates using Dunnett's multiple comparisons test. ∗p ​≤ ​0.05, ∗∗p ​≤ ​0.01, ns ​= ​not significant. (E) 143B cells were transiently transfected with GFP-LAMP1 and exposed to nHA+DOX (50 ​μg/mL) for 4 ​h. Accumulation and co-localization (yellow) of nHA+DOX (red) in GFP-LAMP1 (green) positive lysosomes was detected by confocal microscopy. Arrowheads (white) indicate co-localization of DOX and GFP-LAMP1. (F) Fluorescence intensity profiles along the dotted white line from panel (E) was used for the calculation of Pearson correlation co-efficient. (G, top) Transmission electron microscopy micrograph shows healthy lysosomes with dense round shape. (G, bottom) The accumulation of nHA+DOX in endolysosomes. (H) Release of DOX from nHA+DOX is pH- and time-dependent. (I) Molecular dynamics data showing possible DOX-HA interaction at physiological pH (left) and at lysosomal pH (right).

To investigate the cellular distribution of nHA+DOX, 143B cells were treated with increasing concentration of nHA+DOX (Fig. 4A). By confocal microscopy we observed a concentration dependent increase in cellular uptake of nHA+DOX. In addition, DOX was mainly accumulated in the cytoplasmic vesicles and filamentous structures. Importantly we failed to detect the presence of DOX in tumor cell nuclei (Fig. 4A). The concentration dependent increase in cellular uptake of nHA+DOX was further confirmed by flow cytometry analysis (Fig. 4B).

To investigate the role of endocytic pathways in cellular uptake of nHA+DOX, we used a panel of endocytosis inhibitors (Fig. 4C and D). 143B cells were pretreated with the respective inhibitor for 30 ​min, followed by treatment with nHA+DOX (50 ​μg/mL) for 24 ​h. Cellular uptake of DOX bound to nHA was quantified by flow cytometry. We first investigated the effect of macropinocytosis inhibitors, the Na^+^/H^+^ exchange inhibitor, Amiloride that significantly blocked the cellular uptake of nHA+DOX. The process of macropinocytosis is mainly regulated by PI3 kinase (PI3K). We therefore, tested the effect of PI3K inhibitor on cellular uptake of nHA+DOX. We observed that the PI3K inhibitor, LY294002 significantly blocked the cellular uptake of DOX bound to nHA when compared to nHA+DOX treated cells without inhibitors (Fig. 4C and D), suggesting that PI3K activity plays an important role in cellular uptake of nHA+DOX. Moreover, the potential role of caveolin-mediated endocytosis of nHA+DOX was investigated using methyl-β-cyclodextrin (MβCD), a cholesterol depletion molecule. Similar to other endocytosis inhibitors, MβCD also caused significant decrease in the cellular uptake of DOX bound to nHA. Finally, we tested the potential role of dynamin dependent endocytosis in cellular uptake of nHA+DOX by pretreating the 143B cells with the inhibitor Dynasore, a potent dynamin inhibitor. Dynasore had moderate effect on the cellular uptake of nHA+DOX (Fig. 4C and D). Importantly all the endocytosis inhibitors tested caused significant decrease in the cellular uptake of nHA+DOX (Fig. 4C and D). Together the results suggest that cellular uptake of nHA+DOX is mainly via i) macropinocytosis ii) caveolin and iii) dynamin dependence.

To address if nHA+DOX accumulates in specific intracellular compartment of tumor cells, GFP-LAMP1 transiently transfected 143 ​B cells were treated with nHA+DOX (50 ​μg/mL) for 4 ​h and visualized live by confocal microscopy (Fig. 4E). We observed co-localization of nHA+DOX and GFP-LAMP1, suggesting that nHA+DOX accumulates in tumor cell lysosomes (Fig. 4E and F). TEM images showed that nHA+DOX could penetrate cell membranes as it was found inside the osteosarcoma cells. nHA loaded with DOX were inside the cytoplasm, especially in the lysosome with small rod shape (diameter 20–50 ​nm) (Fig. 4G). TEM ​+ ​EDX images showed that endocytosed nHA+DOX presented a crystalline structure and highly expressed calcium element which was not observed in untreated cells, indicating its indeed the HA component (Fig.S6). To determine if the acidic environment of lysosomes causes release of DOX from nHA, we investigated the effect of pH on nHA+DOX. Interestingly, pH modulated the release of DOX from nHA particles. At pH 2.5, 81% DOX was released from nHA within 72 ​h and 46% DOX was released at pH 5. The lowest release was seen at pH 7.4 with 16% of the drug being released within 72 ​h (Fig. 4H). For mHA+DOX, which are not able to enter the cell (Fig.S7), the acidic pH in the tumor microenvironment (pH 5.6–6.8) caused 8–16% of the drug to be released extracellularly compared to physiological pH (Fig.S8). Molecular dynamics data suggested pH-dependent release of DOX is plausible to be due to the change of protonation states of the functional groups of DOX and the phosphoric acid protonation state that undergoes chemical changes from physiological pH to lysosomal pH. The difference in binding free-energy of the DOX–HA complex at pH 7.4 and pH 5 was estimated by free-energy perturbation to 5 ​± ​1 ​kcal/mol in agreement with a pH release. Absolute binding free-energies of both the complexes indicate a repulsion at both physiological pH and lysosomal pH (Fig. 4I).

#### 3.2.2 nHA mediated delivery of DOX to mitochondria causes mitochondrial dysfunction and inhibition of tumor cell migration

**Fig. 5:**
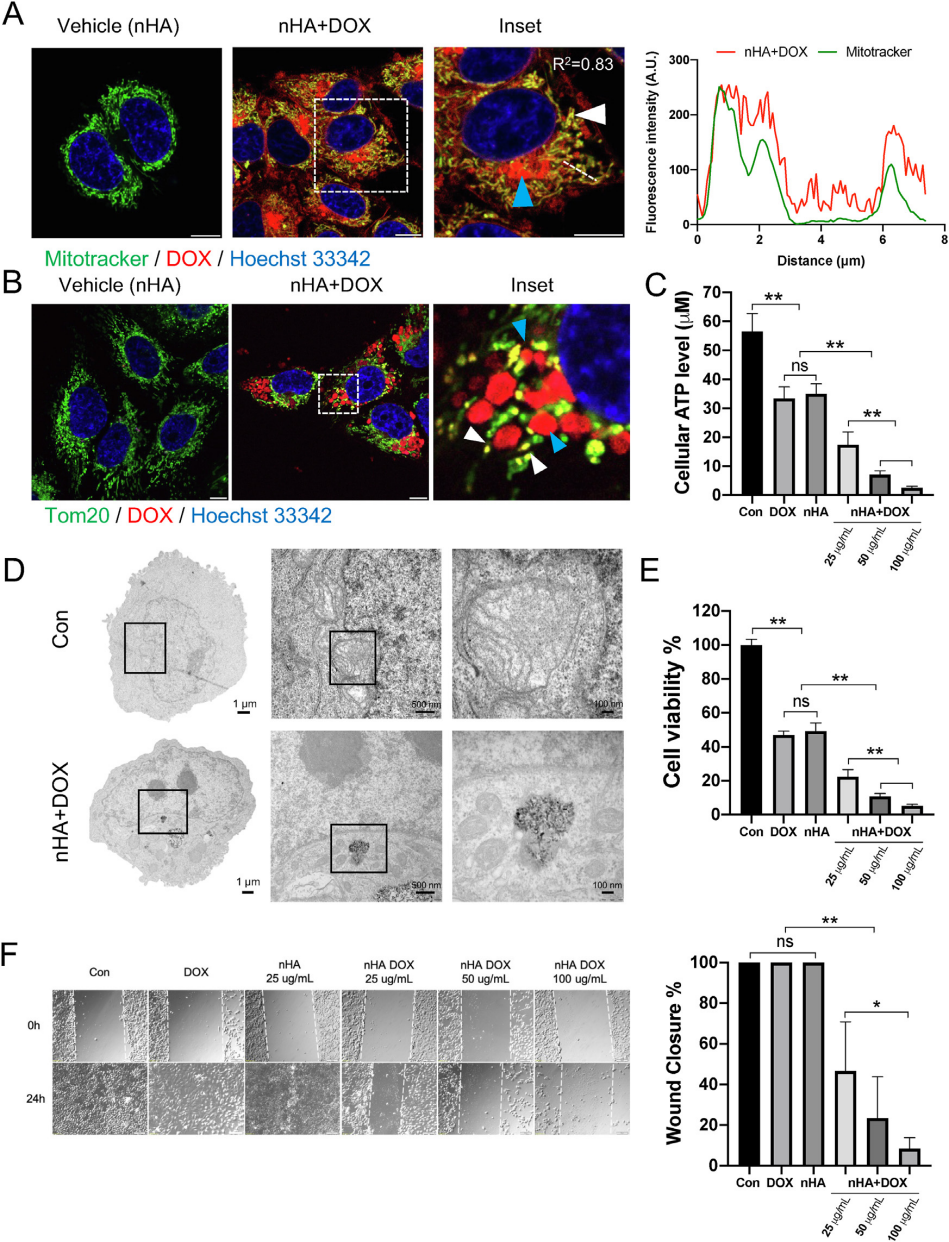
nHA mediated delivery of DOX to mitochondria causes mitochondrial fragmentation and reduction in tumor cell migration. (A–B) 143B cells treated with nHA+DOX (50 ​μg/mL) for 4 ​h, cells were counterstained with mitochondrial marker (green), Mitotracker (500 ​nM) and nuclei marker (blue), Hoechst 33342 (2 ​μM). Arrowhead (white) indicates co-localization of Miotracker (green) with DOX (red), while arrowhead (cyan) indicates perinuclear accumulation of DOX. Line graph to the right indicates fluorescence intensity profile (across white dotted line in left panel) used for calculation of Pearson correlation co-efficient. (B) 143 ​B cells treated with nHA+DOX (50 ​μg/mL) for 24 ​h were stained for mitochondria marker, Tom20 (green). Nuclei of the cells were counterstained for Hoechst 33342 (2 ​μM). Arrowheads (white) shows fragmentation of mitochondria while arrowheads (cyan) show perinuclear accumulation of DOX. (C) Reduction in ATP activity was detected in 143B cells after being treated with nHA+DOX at different concentrations (25–100 ​μg/mL) for 24 ​h. The equivalent DOX concentrations are 0.575, 1.15 and 2.3 ​μg/mL (D) TEM verified the co-localization of nHA+DOX and mitochondria. (E) nHA+DOX leads to more pronounced cell death compared to non-treated, DOX and only nHA group at various concentrations. (F) Bright field images from a wound healing assay showing human osteosarcoma cells have reduced migration ability after being treated by nHA+DOX. One-way ANOVA with Tukey's multiple comparisons test was used to test the difference in cell viability, cell migration and intracellular ATP level. ∗ indicates p ​< ​0.05, ∗∗ indicates p ​< ​0.01, ns indicates not significant.

DOX primarily accumulates in tumor cell nuclei and mitochondria [30]. DOX delivered via nHA was more efficient compared to free DOX. Furthermore, no DOX was found in the cell nucleus when delivered using nHA (Fig.S9). To address if the nHA+DOX delivers DOX to the mitochondria of the cells, 143B cells were treated with nHA+DOX (50 ​μg/mL) for 4 ​h and counterstained with mitochondrial marker, Mitotracker. Interestingly majority of the cytoplasmic pool of DOX was accumulated in the mitochondria of the cell, as shown by co-localization of DOX with Mitotracker (Fig. 5A). Importantly accumulation of DOX in mitochondria led to mitochondrial fragmentation (Fig. 5B). To address if mitochondrial fragmentation may lead to dysfunction of mitochondria, we treated 143B cells with increasing concentration of nHA+DOX for 24 ​h and cellular ATP levels were quantified. We observed concentration dependent decrease in cellular ATP content (Fig. 5C), suggesting that nHA+DOX causes dysfunction of mitochondria. Importantly, nHA+DOX caused rapid decrease in cellular ATP levels compared to nHA or free DOX (Fig. 5C). TEM images demonstrated healthy mitochondria with clear ruffled microstructure of the mitochondrion in untreated cells (Fig. 5D, top panel). However, nHA loaded with DOX were found inside or around the mitochondria inducing its collapse (Fig. 5D, bottom panel). In comparison to untreated cells or cells treated with free DOX, mitochondrial insufficiency resulted in a higher, yet dose-dependent cytotoxicity induced by nHA+DOX (Fig. 5E). Furthermore, due to insufficient ATP supply, the cells treated with nHA+DOX exhibited reduced migration compared to the control groups (Fig. 5F). To explore the biosafety of the combination of n/mHA, HA particles at various concentrations were given to 143B cells and MC3T3 cells. It showed that when used at a similar concentration (50 ​μg/mL) as used for the tumor cell line, significantly lower toxic effect of nHA or the combination was seen on the osteoblast cell line compared to the osteosarcoma cells. Furthermore, n/mHA particles were less cytotoxic to MC3T3 compared to nHA alone within the 50–100 ​μg/mL concentration range (Fig.S10).

### 3.3 The efficacy of HA particles delivering DOX locally

#### 3.3.1 In-vivo tumor eradication by local delivery of DOX using nHA, mHA or n/mHA in human osteosarcoma xenograft

**Fig. 6:**
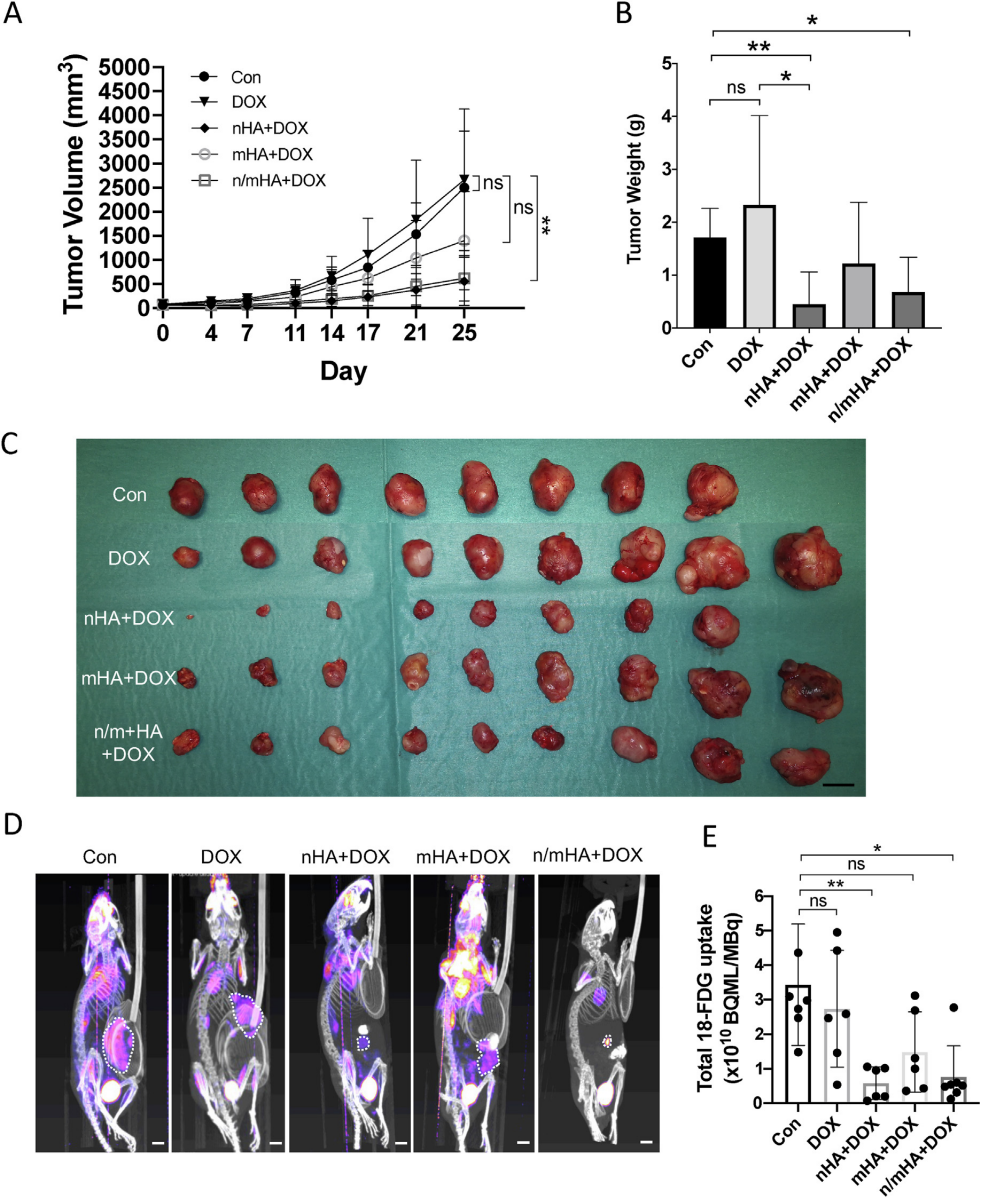
Antitumor efficacy of nHA, mHA or a mixture of nHA/mHA locally delivering DOX in a highly aggressive osteosarcoma model in nude mice. (A) Tumor volume as measured by a Vernier caliper twice a week during the follow-up period. (B) Tumor weight measured for all the samples at the end time point. (C) A photograph of all the tumor tissues collected at the end time point. Scale bar indicates 1 ​cm. (D) Representative PET/CT images from each group with dashed white line indicating the intra-tumoral uptake of ^18^F-FDG. Scale bar indicates 0.5 ​cm. (E) Quantification of the total ^18^F-FDG uptake in the tumor. Kruskal-Wallis test with Dunn's multiple comparisons test was used for comparing treatment groups (G2-G5) to control group (G1) in A, B and E. The intergroup differences in the remaining 4 treatment groups (G2-G5) were further tested separately using Kruskal-Wallis and Dunn's multiple comparisons. ∗ indicates p ​< ​0.05. ∗∗ indicates p ​< ​0.01. ns indicates not significant.

In human xenograft model, DOX I.V injection (G2) had no obvious tumor inhibition compared to no treatment for up to 25 days. nHA+DOX indicated a much stronger tumor inhibition compared to mHA. No obvious difference was observed between nHA and n/mHA (Fig. 6A). Additionally, the tumor weights of the collected tumors verified the efficacy of nHA+DOX and n/mHA+DOX (Fig. 6B). Tumor samples from nHA+DOX (G3) and nano-/micro-HA+DOX (G5) were much smaller than those from control group (G1) and DOX IV group (G2) (Fig. 6C). ^18^F-FDG PET-CT imaging was performed to demonstrate glycolytic activity in different treatment groups as a function of elevated ^18^F-FDG consumption. For mice with no treatment (G1) or DOX IV injection (G2), the tumors were metabolically more active and consumed higher amount of the radioactive deoxy glucose compared with the other three groups (Fig. 6D) (see Fig. S11 for images of all specimens). nHA+DOX group (G3) and n/mHA+DOX group (G5) demonstrated the lowest uptake of ^18^F-FDG in the tumor (Fig. 6E).

#### 3.3.2 Reduced cell proliferation and increased cell apoptosis induced by DOX delivered locally by nHA, mHA or n/m HA moiety

**Fig. 7:**
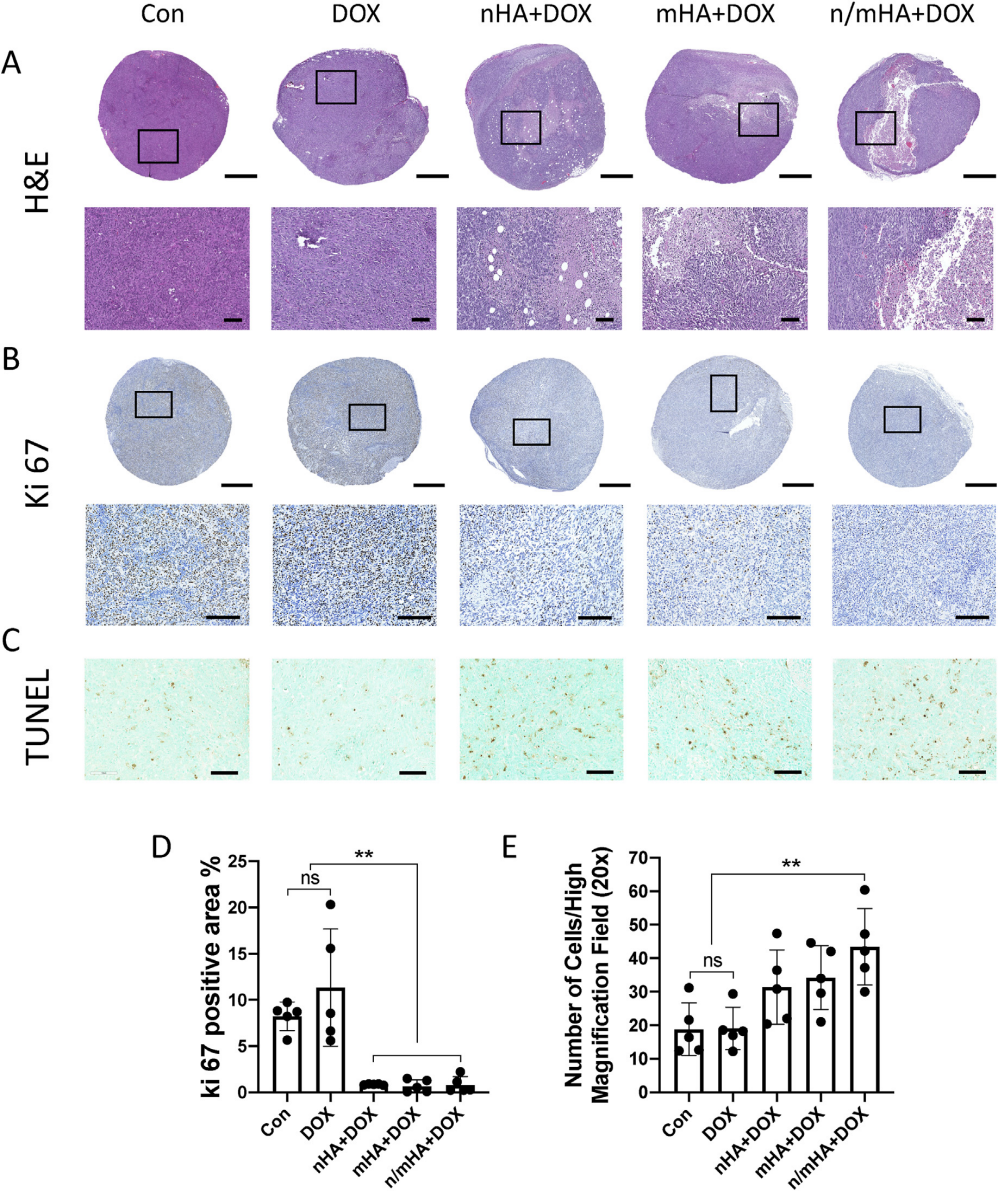
Representative H&E and immunohistochemistry (Ki-67 and TUNEL) staining of samples from each group (G1 to G5). (A) H&E staining showing the microstructure and pathological changes in different treatment groups. Scale bar indicates 800 ​μm for whole section and 100 ​μm for high magnification images. (B) Ki-67 positive cells in the whole section and high magnification images. Scale bar indicates 800 ​μm for overview sections and 200 ​μm for high magnification images. (C) TUNEL positive cells indicating apoptosis in high magnification images. Scale bar indicates 100 ​μm. (D) Quantification of the Ki-67 positive area (n ​= ​5/group). (E) Quantification of the TUNEL positive cells (n ​= ​5/group). One-way ANOVA with Dunnett's multiple comparisons test was used for comparing treatment groups (G2-G5) to the control group (G1). The differences among the other 4 treatment groups (G2-G5) were further tested with one-way ANOVA with Tukey's multiple comparisons. ∗∗ indicates p ​< ​0.01, ns indicates not significant.

In Con (G1) and DOX (G2) group, human osteosarcoma cells were packed tightly in an unorganized pattern. However, there were some widened interstitial space in G3-G5 (Fig. 7A). To detect the cell proliferation within the tumors, Ki 67 was stained for each group (n ​= ​5). Secondary antibody alone staining was taken as negative control, which did not reveal any positive cells in the whole sections (Fig. S12). Compared to Con group (G1), systemic DOX group (G2) had almost similar number of Ki-67 positive cells, which indicated the osteosarcoma cells were highly proliferative in these groups. DOX, when delivered by nano- or micro-HA, dramatically reduced Ki 67 positive cells within the tumor sample (Fig. 7B). The quantification of Ki 67 positive tissue verified the result that G3-G5 significantly reduced cell proliferation compared to G1 and G2 (Fig. 7D). Apoptosis marker TUNEL was also chosen to evaluate the treatment efficacy. dH_2_O, instead of TdT Enzyme, was used as negative control, which showed that no apoptotic cells were found in the whole section (Fig.S13). DOX, delivered by HA particles had more apoptotic cells compared to Con and systemic DOX groups (Fig. 7C). The quantification of TUNEL positive cells per high magnification field verified the result that G3 and G4 had slightly higher cell apoptosis compared to G1 and G2. Furthermore, G5 had significantly increased cell apoptosis compared to G1 and G2 (Fig. 7E).

### 3.4 The mechanism of improved tumor killing effect induced by DOX delivered by HA particles locally compared to systemic injection

**Fig. 8:**
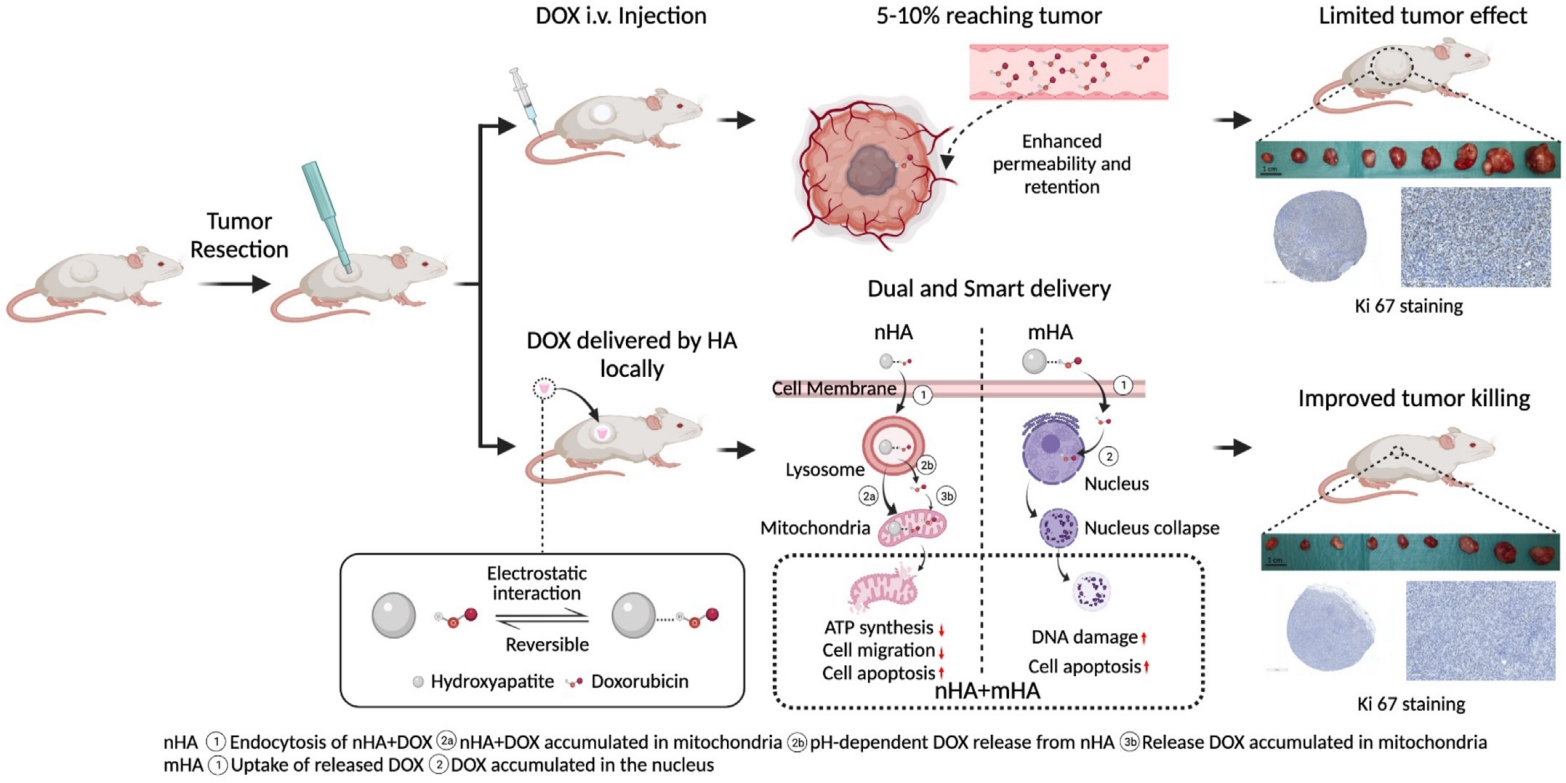
Schematic illustration of the mechanism of DOX delivered by HA locally and its efficacy compared to systemic injection. This figure was made on BioRender.com.

A human osteosarcoma subcutaneous xenograft was replicated in Athymic Nude mice. After the tumor reached an average tumor volume of 72 ​mm^3^ (12 days post cell inoculation), tumor resection was done using a biopsy punch (ø ​= ​2 ​mm) to mimic the clinical situation (Fig. 8, Left panel). Pellets consisting of HA particles (nano-, micro- and combination of both) loaded with DOX were implanted in this defect after tumor resection in the treatment groups. Systemic DOX injection was taken as the positive control group. DOX, when injected intravenously, went through the circulation and 5–10% of the administrated drug was accumulated in the tumor due to the enhanced permeability and retention (EPR) effect. Thus, limited tumor effect was achieved by conventional chemotherapy (Fig. 8, Right top panel). Since DOX could bind to HA (irrespective of the size) and this binding is reversible, it can easily be functionalized with HA particles and delivered based on the particle size (Fig. 8, Right bottom panel). When DOX was delivered by different size of HA particles locally, different drug delivery pathways were found. nHA carrying DOX was endocytosed mainly by i) macropinocytosis ii) caveolin and iii) dynamin dependent. After being entocytosed, nHA+DOX was found accumulated in the lysosome and due to the acidic pH of the lysosome, part of the DOX was released from nHA, which then moved to the mitochondria. The unreleased DOX was carried to mitochondria by nHA. As a result, insufficient ATP synthesis, less cell migration and cell apoptosis were induced. For mHA, DOX was released extracellularly, which passed through the cell membrane and accumulated in the nucleus causing DNA damage and cell apoptosis (Fig. 8, Middle bottom panel). By combing nHA and mHA together, improved tumor killing effect was achieved (Fig. 8, Right bottom panel).

## 4 Discussion

Synthetic HA based bone substitutes have been used during the last few decades to replace damaged bone in trauma, or filling of voids after debriding infected or neoplastic bone tissue [31]. The application of pure HA particles on tumor inhibition, particularly the nano sized particles, is a research field that has gained significant attention recently [32]. In this study, we found that DOX could bind to HA both in-vitro and in-vivo through an electrostatic interaction, irrespective of the size of the HA particles. Being weak, this interaction was easier to disrupt in an acidic microenvironment leading to a pH-dependent drug release without impairing the cytotoxic activity of DOX. Our data showed that nHA carrying DOX enters the cell via endocytosis and is localized in the lysosomes. The acidic pH microenvironment (pH 4.5–5.5) in the lysosomes breaks the DOX-HA interaction with the free DOX being delivered further to the mitochondria. No DOX was seen in the nucleus, which by speculation could be due to the limited amount of free DOX directed towards the nucleus which is likely not sufficient to be detected using the current fluorescence microscopy experimental setup. In an aggressive osteosarcoma model in-vivo, nHA delivering DOX demonstrated a strong tumor eradication effect using the local delivery approach compared to systemic treatment with smaller “metabolically active tumor”. No effect from the systemic administration of DOX compared to control group was observed and might be caused by a relatively lower concentration of DOX being used on an established aggressive tumor. Combining mHA in a carrier with nHA did not compromise the tumor eradication effect but might improve the safety by retaining the nHA locally within the tumor. In the future, based on the in-vivo binding data, retention of pre-implanted HA particles might be used to recruit additional apatite-binding cytostatics to increase the efficacy of the conventional chemotherapy, potentially with fewer side effects.

In this study, HA particles, irrespective of the size, could easily adsorb and form an electrostatic interaction with DOX in a physiologic buffer solution. nHA (<50 ​nm) had a stronger affinity for DOX compared to mHA (10 μm). HA has two binding sites, the C site which is rich in Ca^2+^ and the P site, which is rich in PO_4_^3−^. Both possess affinity towards biological macromolecules such as proteins [33]. DOX has abundant hydroxyl groups in its structure, which makes this interaction with HA easier to form [34]. The molecular simulation indicates that there is an electrostatic interaction between DOX and HA, which is reversible and disrupted under acidic pH. A major difference of 5 ​± ​1 ​kcal/mol in binding free-energy for the DOX–HA complex was seen between pH 7.4 and pH 5, which is in agreement with a pH-dependent release. This was experimentally validated in-vitro both on nHA+DOX and mHA+DOX composites and it was observed that more DOX was released at an acidic pH. This interaction between apatite-binding drug like DOX and HA might also be related to the structure and crystallinity of the material. The physico-chemical properties of HA such as the porosity of the HA material or the degree of crystallinity can also provide further accessible surfaces for electrostatic interaction affecting the drug loading [35]. Since this binding is reversible and controllable, a “switch” for smart delivery of apatite-binding drugs is created. There are several factors which could affect this reversible interaction and make DOX loaded HA a choice for controlled DOX release. For example, DOX-HA interaction is very sensitive to acidic microenvironment under which this interaction breaks and the drug will be released [36]. Previous studies have confirmed that the tumor microenvironment is acidic (pH 5.6 to 6.8) [37], which can lead to an increased drug release in the tumor compared to normal tissues. It can also be affected by the ions around the HA [38], which gives the possibility to control the drug delivery based on the ionic distribution around the HA-DOX complex [39]. Ultrasonic irradiation has also been shown to break this type of chemical interaction, which provides the possibility to control the drug release by providing external physical stimuli [40]. Except cytostatics, the chemical interactions like hydrogen bonding have also been used in self-assembled construction of nanophotosensitizers for cancer with longer local retention and tumor killing effect [41,42]. Similar interactions of other biomolecules, particularly 3rd generation bisphosphonates like zoledronic acid, known to bind to HA have been widely used systemically for bone tumor inhibition [43]. HA-binding antimicrobial peptides have been designed for bone infections [44]. Thus, the chemical structure of various drugs could be chemically modulated based on the HA binding sites for targeted delivery in bone disorders.

The in-vitro DOX-HA interaction was also verified by the in-vivo muscle pouch model in which we confirmed that HA can be used as a moiety for adsorbing systemically circulating apatite-binding cytostatics like DOX. Interestingly, significantly more DOX was found in the HA materials than in a collagen scaffold or in the surrounding muscle. This recruitment of circulating DOX is a consequence of progressive vascularization at the tissue-implant interface between day 2 and 10 after implantation. Due to the high expression of vascular endothelial factor (VEGF), and an intricate vascular network forming around the material, circulating drugs with possible affinity to HA thereby could reach the material [45]. After being injected systemically as per the clinical protocol, DOX will accumulate in various organs through the circulation, such as the liver, kidney, lung and heart [46]. By implanting HA particles before drug administration, more DOX can be recruited to the HA than to the nearby tissue like the surrounding muscle and other organs, potentially alleviating the off-target side effects. Other drugs have also been shown to be effective in animal models. For instance, zoledronic acid, when systemically injected, could seek pre-implanted hydroxyapatite particles and induce more bone formation around an implant [7,22]. Whether recruited DOX in HA will actually have a tumor inhibiting effect needs to be explored further in an in-vivo tumor model.

Previous studies have shown that cells of different tissue origins can endocytose nanoparticles fabricated from a variety of substrate materials provided that their size is in the nanometer range (<1000 ​nm) [47]. It has been confirmed that HA nanoparticles can penetrate cancer cell membranes via endocytosis, eventually inducing apoptosis [48]. Despite a toxic effect of HA particles on tumor cells, the application of pristine HA nanoparticles to eradicate tumors in-vivo has been unsuccessful due to limited cytotoxicity for established tumor [26]. To overcome this limitation, a few studies tried to couple HA particles with paclitaxel by chitosan or bovine serum albumin (BSA) coatings, but a rapid release of paclitaxel was observed within the first 3–5 days together with impaired cytostatic activity of the drug [49]. In this study, DOX was successfully delivered intracellularly by the nHA without impairing its biological activity. nHA+DOX which had been engulfed by the cells, were seen accumulated in lysosome first and then moved to mitochondria instead of the nucleus as is the case with free DOX [50]. Lysosome, which is the intrinsic digestive system of cells, receiving and sequestering cargoes from phagocytosis, endocytosis and auto phagocytosis engulfs the nHA+DOX composite during endocytosis [51]. For cancer cells, endocytosis is a fundamental biological process used to internalize (bio)molecules and nanoparticles [52]. The size of the particles may affect the uptake efficiency and kinetics, the internalization mechanism and also the subcellular distribution [53]. A size-dependent uptake in different cell lines has been observed for various nanoparticles [54,55] with the maximum cellular uptake at a NP core size in the range of 30–50 ​nm. This suggests that there is an optimal size for active uptake. In our study, nHA with less than 50 ​nm was only used for intracellular delivery of DOX. The size and shape of HA nanoparticles for optimal DOX loading and delivery needs to be explored in future studies. For lysosomes, an acidic environment (pH 4.5–5.5) promotes protein degradation during cellular metabolism, which in our case disturbs the DOX-HA interaction, resulting in pH-dependent drug release (72) as verified in-vitro. Various studies have explored the pH-dependent controlled drug delivery from acidic tumor microenvironment and the lysosomes [56]. For example, when delivered by a calcium sulfate/micro hydroxyapatite biomaterial, DOX has been confirmed to have a higher in-vitro release in acidic pH, which mimics the tumor microenvironment [27]. However, few studies have also paid attention to intracellular pH-dependent release of drugs, especially with nanomaterials [57].

Mitochondria are essential intracellular organelles that regulate energy metabolism, cell death, and signaling pathways that are important for cell proliferation and differentiation. Emerging evidence suggests that cancer is primarily a mitochondrial metabolic disease [58]. Mitochondrial function in cancer is the dissemination of tumor cells to distant organs, or metastasis [59], which is responsible for over 90% of cancer deaths [60]. Various biomaterials have been explored to target mitochondria inducing damage and apoptosis [61,62]. In our study, we found that HA nanoparticles, when loaded with DOX, could enter the cancer cell and eventually accumulate in the mitochondria inducing mitochondrial dysfunction and collapse, which results in less cell migration. Mitochondria are semi-autonomous organelles that perform essential functions in cellular metabolism and the regulation of cell death [63]. We found DOX, delivered by nHA, could inhibit the production of ATP after accumulating in the mitochondria, which was significantly reduced with the free drug. As a result, the cancer cell migration was dramatically inhibited due to insufficient ATP synthesis. It even induced the collapse of mitochondria and a stronger cytotoxicity was achieved by mitochondria-targeted delivery of DOX instead of nuclear delivery. Except for ATP production, mitochondria also play an important role in cancer through other energy and macromolecular synthesis, which is related to further metastasis [64]. As a prominent signal for cancer severity, mitochondrial dysfunction shows a significant correlation with poorer tumor progression and decreased metastasis [65]. In the nHA+DOX group, the tumors had the lowest ^18^F-FDG uptake indicating a rather low “metabolic” tumor with lower risk for further metastasis. This could eventually also be an alternative treatment regimen against multidrug resistant solid tumors, which has been an important research field recently [66,67].

The translation of nanomaterials from the bench to bedside has been slow primarily due to concerns regarding safety. The safety of nanomaterials being used in the clinics has always been a concern, primarily related to the distribution of the particles after being administrated. Circulating nanoparticles can induce damage to lung, heart, liver and spleen with a high dose accumulation [68]. Kollenda SA et al. tried to explore the biodistribution of calcium phosphate nanoparticles after repeated administration by PET/CT. When implanted locally within the tumor, almost 90% of the nanoparticles stayed locally with 10% having migrated to the spleen, liver and lung [69]. It must however be noted that the biodistribution study was performed within a short period (5 ​h) due to limitations from radioactive labelling of the nanoparticles. Long-term studies need to be conducted to explore its safety further. To decrease this risk, we attempted to embed the HA nanoparticles together with HA microparticles. The in-vitro data showed that the combination of n/mHA had less cytotoxic effects on healthy osteoblasts compared to nHA alone. The in-vivo data showed no major differences in nHA+DOX group vs. the nHA/mHA+DOX group for tumor killing. Thus, using mHA as a carrier for nHA did not compromise the efficacy of nHA+DOX but might enhance the safety profile of the DOX delivery system. Unlike nHA, mHA (10 ​μm) cannot be taken up by cancer cells [70], but could provide sustained extracellular release of the loaded DOX over a 28-day period [27]. When used as a n/mHA composite in a highly aggressive osteosarcoma, nHA could enable quick intracellular mitochondria-targeted delivery of cytostatics inside cancer cells leading to improved tumor eradication while the mHA could provide a sustained delivery of DOX extracellularly to achieve a long-term tumor inhibition as well as improved retention of HA nanoparticles locally. Simultaneously, mHA will also work as a template for bone formation to repair a bone defect caused by the resection of a bone tumor and stay locally for several months [25]. Furthermore, remaining pre-implanted HA particles might act as a recruiting platform for systemically circulating apatite-binding cytostatics, which could further enhance the efficacy of conventional chemotherapy. The right distribution of the nano/micro combination for sufficient drug recruitment needs to be explored further.

There are a few limitations in this study. Firstly, due to ethical considerations (tumor size in control groups), we had to terminate the in-vivo experiment at day 25. At this time point, we were not able to find any difference between nHA and n/mHA loaded with DOX. Secondly, we fabricated the particles into pellets using 10 ​mg/mL hyaluronic acid before implantation, which may have hindered the dissemination of the particles within the tumor, undermining the ‘real’ impact of fully exposed HA particles. Additionally, we were unable to decipher the mechanism by which DOX accumulates in mitochondria when given through nHA particles. Further investigation is necessary to determine if this is a nanoparticle-related phenomena or whether the intracellular transport mechanism is changed after intracellular DOX administration.

## 5 Conclusion

We show that DOX, a cornerstone osteosarcoma drug shows accretion to particulate HA of both nano and micro size. Systemically administered DOX seeks and binds HA particles with more binding to nHA compared with mHA. In-vitro cellular interactions with DOX functionalized nHA indicate that the nHA-DOX composite leads to intracellular delivery of DOX, targeting the mitochondria and affecting the consequent downstream cascades leading to reduced cell migration and increased apoptosis. mHA+DOX can extra-cellularly release DOX over a long period of time and by combining the nHA and mHA particles, the biocompatibility of HA particles can be enhanced. By applying the nHA/mHA carrier system delivering DOX for the treatment of a localized osteosarcoma in a mice model, we show a significant reduction in the tumor growth with the use of n/mHA+DOX, in fact better than a systemic DOX treatment regimen. No disruptive new treatment modalities for solid bone tumors have evolved in the last three decades. The approach of locally injecting nano-/micro-HA particles pre-functionalized with a HA binding cytostatic drug-DOX, opens new avenues for complimentary treatment of localised solid tumors. Postoperative systemic reloading with apatite-seeking cytostatics is a completely novel treatment using prior local implanted hydroxyapatite particles, acting as a Trojan horse in a solid tumor.

## Author contributions

DBR, LL, MT and YL: Conception. YL, AN, MO, DBR, SS: In-vitro experiments. YL, DBR, SS, MT, HI: In-vivo study and imaging. YL, AN, MO, SW, ES, JE, HI, DBR, MT and LL: Data analysis. YL: Manuscript draft. All authors: Manuscript revision.

## Conflict of interest

LL is a board member of BoneSupport AB, Sweden and OrthoCell, Australia.

LL, MT, DBR and YL hold stocks in Moroxite AB, Sweden.

## Declaration of competing interest

The authors declare the following financial interests/personal relationships which may be considered as potential competing interests: Lars Lidgren reports a relationship with BoneSupport AB that includes: board membership. Lars Lidgren reports a relationship with Orthocell Limited that includes: board membership. Lars Lidgren reports a relationship with Moroxite AB that includes: board membership and equity or stocks. Magnus Tagil reports a relationship with Moroxite AB that includes: board membership and equity or stocks. Deepak Bushan Raina reports a relationship with Moroxite AB that includes: board membership and equity or stocks. Yang Liu reports a relationship with Moroxite AB that includes: equity or stocks.

## References

[1] R. Forsyth, P.C.W. Hogendoorn, Chapter 2 - epidemiology of primary bone tumors and economical aspects of bone metastases, in: D. Heymann (Ed.), Bone Sarcomas and Bone Metastases - from Bench to Bedside (third ed.), Academic Press2022, pp. 17-23.

[2] Bielack S.S., Kempf-Bielack B., Delling G., Exner G.U., Flege S., Helmke K., Kotz R., Salzer-Kuntschik M., Werner M., Winkelmann W., Zoubek A., Jürgens H., Winkler K. (2002). Prognostic factors in high-grade osteosarcoma of the extremities or trunk: an analysis of 1,702 patients treated on neoadjuvant cooperative osteosarcoma study group protocols. J. Clin. Oncol..

[3] Marina N.M., Smeland S., Bielack S.S., Bernstein M., Jovic G., Krailo M.D., Hook J.M., Arndt C., van den Berg H., Brennan B., Brichard B., Brown K.L.B., Butterfass-Bahloul T., Calaminus G., Daldrup-Link H.E., Eriksson M., Gebhardt M.C., Gelderblom H., Gerss J., Goldsby R., Goorin A., Gorlick R., Grier H.E., Hale J.P., Hall K.S., Hardes J., Hawkins D.S., Helmke K., Hogendoorn P.C.W., Isakoff M.S., Janeway K.A., Jürgens H., Kager L., Kühne T., Lau C.C., Leavey P.J., Lessnick S.L., Mascarenhas L., Meyers P.A., Mottl H., Nathrath M., Papai Z., Randall R.L., Reichardt P., Renard M., Safwat A.A., Schwartz C.L., Stevens M.C.G., Strauss S.J., Teot L., Werner M., Sydes M.R., Whelan J.S. (2016). Comparison of MAPIE versus MAP in patients with a poor response to preoperative chemotherapy for newly diagnosed high-grade osteosarcoma (EURAMOS-1): an open-label, international, randomised controlled trial. Lancet Oncol..

[4] Hanafy E., Al Jabri A., Gadelkarim G., Dasaq A., Nazim F., Al Pakrah M. (2018). Tumor histopathological response to neoadjuvant chemotherapy in childhood solid malignancies: is it still impressive?. J. Invest. Med..

[5] Pizzoccaro M.A., Nikel O., Sene S., Philippe C., Mutin P.H., Bégu S., Vashishth D., Laurencin D. (2016). Adsorption of benzoxaboroles on hydroxyapatite phases. Acta Biomater..

[6] LeGeros R.Z. (2008). Calcium phosphate-based osteoinductive materials. Chem. Rev..

[7] Raina D.B., Liu Y., Isaksson H., Tägil M., Lidgren L. (2020). Synthetic hydroxyapatite: a recruiting platform for biologically active molecules. Acta Orthop..

[8] Bauer I.W., Li S.P., Han Y.C., Yuan L., Yin M.Z. (2008). Internalization of hydroxyapatite nanoparticles in liver cancer cells. J. Mater. Sci. Mater. Med..

[9] Chen X., Deng C., Tang S., Zhang M. (2007). Mitochondria-dependent apoptosis induced by nanoscale hydroxyapatite in human gastric cancer SGC-7901 cells. Biol. Pharm. Bull..

[10] Qing F., Wang Z., Hong Y., Liu M., Guo B., Luo H., Zhang X. (2012). Selective effects of hydroxyapatite nanoparticles on osteosarcoma cells and osteoblasts. J. Mater. Sci. Mater. Med..

[11] Zhang K., Zhou Y., Xiao C., Zhao W., Wu H., Tang J., Li Z., Yu S., Li X., Min L., Yu Z., Wang G., Wang L., Zhang K., Yang X., Zhu X., Tu C., Zhang X. (2019). Application of hydroxyapatite nanoparticles in tumor-associated bone segmental defect. Sci. Adv..

[12] Hou C.H., Hou S.M., Hsueh Y.S., Lin J., Wu H.C., Lin F.H. (2009). The in vivo performance of biomagnetic hydroxyapatite nanoparticles in cancer hyperthermia therapy. Biomaterials.

[13] Hou G., Zhou F., Tian Y., Ji H., Zhang Z., Guo Y., Lv Y. (2014). Predicting the need for blood transfusions in elderly patients with pertrochanteric femoral fractures. Injury.

[14] Yanhua W., Hao H., Li Y., Zhang S. (2016). Selenium-substituted hydroxyapatite nanoparticles and their in vivo antitumor effect on hepatocellular carcinoma. Colloids Surf. B Biointerfaces.

[15] Koski C., Vu A.A., Bose S. (2020). Effects of chitosan-loaded hydroxyapatite on osteoblasts and osteosarcoma for chemopreventative applications, Materials science & engineering. C, Materials for biological applications.

[16] Liu Y., Qiao Z., Gao J., Wu F., Sun B., Lian M., Qian J., Su Y., Zhu X., Zhu B. (2021). Hydroxyapatite-bovine serum albumin-paclitaxel nanoparticles for locoregional treatment of osteosarcoma. Adv Healthc Mater.

[17] Wang N., Cheng X., Li N., Wang H., Chen H. (2019). Nanocarriers and their loading strategies. Adv Healthc Mater.

[18] Borum-Nicholas L., Wilson O.C. (2003). Surface modification of hydroxyapatite. Part I. Dodecyl alcohol. Biomaterials.

[19] Hilbrig F., Freitag R. (2012).

[20] Hossain M., Irwin R., Baumann M.J., McCabe L.R. (2005). Hepatocyte growth factor (HGF) adsorption kinetics and enhancement of osteoblast differentiation on hydroxyapatite surfaces. Biomaterials.

[21] Alt V., Pfefferle H.-J., Kreuter J., Stahl J.-P., Pavlidis T., Meyer C., Mockwitz J., Wenisch S., Schnettler R. (2004). Effect of glycerol-l-lactide coating polymer on bone ingrowth of bFGF-coated hydroxyapatite implants. J. Contr. Release.

[22] Raina D.B., Larsson D., Sezgin E.A., Isaksson H., Tägil M., Lidgren L. (2019). Biomodulation of an implant for enhanced bone-implant anchorage. Acta Biomater..

[23] Wang X., Zhong X., Li J., Liu Z., Cheng L. (2021). Inorganic nanomaterials with rapid clearance for biomedical applications. Chem. Soc. Rev..

[24] Kolmas J., Krukowski S., Laskus A., Jurkitewicz M. (2016). Synthetic hydroxyapatite in pharmaceutical applications. Ceram. Int..

[25] Amini A.R., Laurencin C.T., Nukavarapu S.P. (2012). Bone tissue engineering: recent advances and challenges. Crit. Rev. Biomed. Eng..

[26] Patra J.K., Das G., Fraceto L.F., Campos E.V.R., Rodriguez-Torres M.D.P., Acosta-Torres L.S., Diaz-Torres L.A., Grillo R., Swamy M.K., Sharma S., Habtemariam S., Shin H.-S. (2018). Nano based drug delivery systems: recent developments and future prospects. J. Nanobiotechnol..

[27] Liu Y., Raina D.B., Sebastian S., Nagesh H., Isaksson H., Engellau J., Lidgren L., Tägil M. (2021). Sustained and controlled delivery of doxorubicin from an in-situ setting biphasic hydroxyapatite carrier for local treatment of a highly proliferative human osteosarcoma. Acta Biomater..

[28] Raina D.B., Isaksson H., Hettwer W., Kumar A., Lidgren L., Tägil M. (2016). A biphasic calcium sulphate/hydroxyapatite carrier containing bone morphogenic protein-2 and zoledronic acid generates bone. Sci. Rep..

[29] Schindelin J., Arganda-Carreras I., Frise E., Kaynig V., Longair M., Pietzsch T., Preibisch S., Rueden C., Saalfeld S., Schmid B., Tinevez J.Y., White D.J., Hartenstein V., Eliceiri K., Tomancak P., Cardona A. (2012). Fiji: an open-source platform for biological-image analysis. Nat. Methods.

[30] Cova D., De Angelis L., Monti E., Piccinini F. (1992). Subcellular distribution of two spin trapping agents in rat heart: possible explanation for their different protective effects against doxorubicin-induced cardiotoxicity. Free Radic. Res. Commun..

[31] Wang W., Yeung K.W.K. (2017). Bone grafts and biomaterials substitutes for bone defect repair: a review. Bioact Mater.

[32] Verné E., Bruno M., Miola M., Maina G., Bianco C., Cochis A., Rimondini L. (2015). Composite bone cements loaded with a bioactive and ferrimagnetic glass-ceramic: leaching, bioactivity and cytocompatibility. Mater Sci Eng C Mater Biol Appl.

[33] Kandori K., Toshima S., Wakamura M., Fukusumi M., Morisada Y. (2010). Effects of modification of calcium hydroxyapatites by trivalent metal ions on the protein adsorption behavior. J. Phys. Chem. B.

[34] Hutchins K.M. (2018). Functional materials based on molecules with hydrogen-bonding ability: applications to drug co-crystals and polymer complexes. R. Soc. Open Sci..

[35] Shen S., Wu Y., Liu Y., Wu D. (2017). High drug-loading nanomedicines: progress, current status, and prospects. Int. J. Nanomed..

[36] Zhuo S., Zhang F., Yu J., Zhang X., Yang G., Liu X. (2020). pH-sensitive biomaterials for drug delivery. Molecules.

[37] Chiche J., Brahimi-Horn M.C., Pouysségur J. (2010). Tumour hypoxia induces a metabolic shift causing acidosis: a common feature in cancer. J. Cell Mol. Med..

[38] Shang S., Zhao Q., Zhang D., Sun R., Tang Y. (2019). Molecular dynamics simulation of the adsorption behavior of two different drugs on hydroxyapatite and Zn-doped hydroxyapatite. Mater Sci Eng C Mater Biol Appl.

[39] Zhao D., Yang N., Wei Y., Jin Q., Wang Y., He H., Yang Y., Han B., Zhang S., Wang D. (2020). Sequential drug release via chemical diffusion and physical barriers enabled by hollow multishelled structures. Nat. Commun..

[40] Mitchell M.J., Billingsley M.M., Haley R.M., Wechsler M.E., Peppas N.A., Langer R. (2021). Engineering precision nanoparticles for drug delivery. Nat. Rev. Drug Discov..

[41] Jiang S., Xiao M., Sun W., Crespy D., Mailänder V., Peng X., Fan J., Landfester K. (2020). Synergistic anticancer therapy by ovalbumin encapsulation-enabled tandem reactive oxygen species generation. Angew Chem. Int. Ed. Engl..

[42] Xi D., Xu N., Xia X., Shi C., Li X., Wang D., Long S., Fan J., Sun W., Peng X. (2021). Strong π-π stacking stabilized nanophotosensitizers: improving tumor retention for enhanced therapy for large tumors in mice. Adv Mater.

[43] Park Y.E., Bava U., Lin J.M., Cornish J., Naot D., Reid I.R. (2019). Bone-bound bisphosphonates inhibit proliferation of breast cancer cells. Calcif. Tissue Int..

[44] Huang Z.B., Shi X., Mao J., Gong S.Q. (2016). Design of a hydroxyapatite-binding antimicrobial peptide with improved retention and antibacterial efficacy for oral pathogen control. Sci. Rep..

[45] Gustafsson T. (2011). Vascular remodelling in human skeletal muscle. Biochem. Soc. Trans..

[46] Rahman A., Carmichael D., Harris M., Roh J.K. (1986). Comparative pharmacokinetics of free doxorubicin and doxorubicin entrapped in cardiolipin liposomes. Cancer Res..

[47] Jeevanandam J., Barhoum A., Chan Y.S., Dufresne A., Danquah M.K. (2018). Review on nanoparticles and nanostructured materials: history, sources, toxicity and regulations. Beilstein J. Nanotechnol..

[48] Behzadi S., Serpooshan V., Tao W., Hamaly M.A., Alkawareek M.Y., Dreaden E.C., Brown D., Alkilany A.M., Farokhzad O.C., Mahmoudi M. (2017). Cellular uptake of nanoparticles: journey inside the cell. Chem. Soc. Rev..

[49] Liu Y., Qiao Z., Gao J., Wu F., Sun B., Lian M., Qian J., Su Y., Zhu X., Zhu B. (2020). Hydroxyapatite-bovine serum albumin-paclitaxel nanoparticles for locoregional treatment of osteosarcoma. Adv Healthc Mater.

[50] Gigli M., Doglia S.M., Millot J.M., Valentini L., Manfait M. (1988). Quantitative study of doxorubicin in living cell nuclei by microspectrofluorometry. Biochim. Biophys. Acta Gene Struct. Expr..

[51] Zhitomirsky B., Assaraf Y.G. (2016). Lysosomes as mediators of drug resistance in cancer. Drug Resist. Updates.

[52] Iversen T.-G., Skotland T., Sandvig K. (2011). Endocytosis and intracellular transport of nanoparticles: present knowledge and need for future studies. Nano Today.

[53] Jiang W., Kim B.Y., Rutka J.T., Chan W.C. (2008). Nanoparticle-mediated cellular response is size-dependent. Nat. Nanotechnol..

[54] Chithrani B.D., Ghazani A.A., Chan W.C. (2006). Determining the size and shape dependence of gold nanoparticle uptake into mammalian cells. Nano Lett..

[55] Varela J.A., Bexiga M.G., Åberg C., Simpson J.C., Dawson K.A. (2012). Quantifying size-dependent interactions between fluorescently labeled polystyrene nanoparticles and mammalian cells. J. Nanobiotechnol..

[56] Chen B., Dai W., He B., Zhang H., Wang X., Wang Y., Zhang Q. (2017). Current multistage drug delivery systems based on the tumor microenvironment. Theranostics.

[57] Meng F., Cheng R., Deng C., Zhong Z. (2012). Intracellular drug release nanosystems. Mater. Today.

[58] Vyas S., Zaganjor E., Haigis M.C. (2016). Mitochondria and cancer. Cell.

[59] Pienta K.J., Robertson B.A., Coffey D.S., Taichman R.S. (2013). The cancer diaspora: metastasis beyond the seed and soil hypothesis. Clin. Cancer Res..

[60] Steeg P.S. (2016). Targeting metastasis. Nat. Rev. Cancer.

[61] Zhu Y.X., Jia H.R., Gao G., Pan G.Y., Jiang Y.W., Li P., Zhou N., Li C., She C., Ulrich N.W., Chen Z., Wu F.G. (2020). Mitochondria-acting nanomicelles for destruction of cancer cells via excessive mitophagy/autophagy-driven lethal energy depletion and phototherapy. Biomaterials.

[62] Shen J., Rees T.W., Zhou Z., Yang S., Ji L., Chao H. (2020). A mitochondria-targeting magnetothermogenic nanozyme for magnet-induced synergistic cancer therapy. Biomaterials.

[63] Carew J.S., Huang P. (2002). Mitochondrial defects in cancer. Mol. Cancer.

[64] Phan L.M., Yeung S.-C.J., Lee M.-H. (2014). Cancer metabolic reprogramming: importance, main features, and potentials for precise targeted anti-cancer therapies. Cancer Biol Med.

[65] Porporato P.E., Filigheddu N., Pedro J.M.B., Kroemer G., Galluzzi L. (2018). Mitochondrial metabolism and cancer. Cell Res..

[66] Liu J., Zhu C., Xu L., Wang D., Liu W., Zhang K., Zhang Z., Shi J. (2020). Nanoenabled intracellular calcium bursting for safe and efficient reversal of drug resistance in tumor cells. Nano Lett..

[67] Jin R., Liu Z., Liu T., Yuan P., Bai Y., Chen X. (2021). Redox-responsive micelles integrating catalytic nanomedicine and selective chemotherapy for effective tumor treatment. Chin. Chem. Lett..

[68] Hussain S., Vanoirbeek J.A., Haenen S., Haufroid V., Boland S., Marano F., Nemery B., Hoet P.H. (2013). Prior lung inflammation impacts on body distribution of gold nanoparticles. BioMed Res. Int..

[69] Kollenda S.A., Klose J., Knuschke T., Sokolova V., Schmitz J., Staniszewska M., Costa P.F., Herrmann K., Westendorf A.M., Fendler W.P., Epple M. (2020). In vivo biodistribution of calcium phosphate nanoparticles after intravascular, intramuscular, intratumoral, and soft tissue administration in mice investigated by small animal PET/CT. Acta Biomater..

[70] Paul M.B., Stock V., Cara-Carmona J., Lisicki E., Shopova S., Fessard V., Braeuning A., Sieg H., Böhmert L. (2020). Micro- and nanoplastics – current state of knowledge with the focus on oral uptake and toxicity. Nanoscale Advances.

