# Supplementary material for: Bone mineral: A trojan horse for bone cancers. Efficient mitochondria targeted delivery and tumor eradication with nano hydroxyapatite containing doxorubicin
Source: Mater Today Bio. 2022 Feb 26;14:100227. doi: 10.1016/j.mtbio.2022.100227 (PMC8898975; doi:10.1016/j.mtbio.2022.100227)
Supplement: Multimedia component 1 [file mmc1.docx]

Supplementary Materials for

**Bone mineral: A trojan horse for bone cancers**

**Efficient mitochondria targeted delivery and tumor eradication with nano hydroxyapatite containing doxorubicin**

Yang Liu, Aftab Nadeem, Sujeesh Sebastian, Martin A. Olsson, Sun N. Wai, Emelie Styring, Jacob Engellau, Hanna Isaksson, Magnus Tägil, Lars Lidgren, Deepak Bushan Raina

*Corresponding author. Email: [liu.yang@med.lu.se](mailto:liu.yang@med.lu.se)

**This PDF file includes:**

Supplementary Text

Figs. S1 to S13 (Page 2-14)

Method for Molecular dynamics calculation (Page 15-19)

Supplementary Figure S1. Size distribution for HA microparticles and nanoparticles by TEM. TEM images showed that HA microparticles presented a sphere shape with diameter around 1-10 μm. nHA presented in a rod-like shape with diameter around 20-50 nm.

Supplementary Figure S2. Verification of nHA by TEM+EDS. TEM images showed that HA nanoparticles showed a rod-like shape with diameter around 20-50 nm. XEDS showed that HA nanoparticles with high density in TEM (pink box) exhibited peaks for Ca and P. Scale bar=100 nm.

**Supplementary Figure S3. The effect of protein passivation of HA with FBS on DOX-HA binding.** The bar graph shows that the pre-treatment of nHA particles with FBS reduced the DOX binding to HA by only 7%.

Supplementary Figure S4. All the samples collected from the DOX-HA in-vivo binding experiment. These samples were scanned by IVIS Spectrum In Vivo Imaging System (IVIS Spectrum CT) for epi-fluorescence. Bright yellow color indicates high fluorescence while dark red indicates low fluorescence. D in the control group for mHA indicates animal death.

Supplementary Figure S5. DOX binding to HA materials or surrounding muscle showed a dose-dependent trend for all groups. One-way ANOVA with Tukey's multiple comparisons test or Kruskal-Wallis and Dunn’s multiple comparisons test was used to detect the difference between different injected DOX doses for each material or only muscle. The dark line with * represents the comparison within only nHA material injected with various DOX doses. The blue line with * represents the comparison within only mHA material injected with various DOX doses. The green line with * represents the comparison within only collagen material injected with various DOX doses. The turquoise line with * represents the comparison within only surrounding muscle injected with various DOX doses. * indicates P<0.05, ** indicates P<0.01, *** indicates P<0.001, **** indicates 0.0001.

**Supplementary Figure S6. Cellular uptake of nHA+DOX was further verified by TEM+EDS.** TEM images showed that no HA particles were present in control cells and a nominal Ca peak in the XEDS spectra was found in the pink box indicating intracellular calcium. For cells treated with nHA+DOX, high density rod-like particles were found inside the cells and prominent Ca and P peaks could be detected in the XEDS spectrum when compared to the background.

**Supplementary Figure S7. Cellular uptake of mHA evaluated by TEM.** The representative TEM images showed that > 95% of the screened cells did not endocytose the mHA particles. Out of all the observed cells (30-40 specimens in total), only one cell indicated the presence of a HA like dark material inside the cell, which is speculated to be a fragmented mHA particle.

**Supplementary Figure S8. Release of DOX from mHA+DOX is pH- and time-dependent.** The pH dependent release was studied under 3 different pH (pH 5.6, 6.8 and 7.4) conditions.

**Supplementary Figure S9. nHA delivers DOX more efficiently compared to free DOX.** (A) 143B cells were treated with nHA bound DOX or free DOX for 24 h. Cellular uptake of DOX was quantified by flow cytometry analysis. Histogram indicates quantification of DOX cellular uptake. Data are representative of two independent experiments; bar graphs show Mean ± SD. Significance was determined from replicates using a one-way analysis of variance (ANOVA) with Sidak’s post-test. * indicates p<0.05, ** indicates p<0.01. (B) 143B cells were treated with nHA bound DOX or free DOX for 24 h and cellular distribution of DOX was investigated by confocal microscopy. Arrowheads (white) indicates accumulation of DOX in cytoplasmic vesicles, while arrowhead (yellow) indicates nuclear localization of DOX. The cyan arrowhead shows accumulation of DOX in tubular structures. Scale bar=10 μm.

**Supplementary Figure S10. The cytotoxicity of pristine HA particles on 143B cells and MC3T3 cells. (A)** Shows they cytotoxic effect of nHA, mHA or the combination of n/mHA on 143B osteosarcoma cells. (B) Shows the cytotoxic effects of nHA or the combination of nHA/mHA on osteoblast cell line MC3T3. Note that the concentration matched HA particles have higher cytotoxic effects on the tumor cells compared with the osteoblasts. Furthermore, n/mHA is more biocompatible with MC3T3 cells compared to nHA alone under 50-100 μg/mL concentrations.

**Supplementary Figure S11. PET-CT images for all animals from each group being scanned at day 20 post-operation.** The white dashed line indicates “metabolically active tumor”. Scale bar indicates 0.5 cm.

**Supplementary Figure S12. Specificity of immunohistochemistry staining (Ki 67).** All the stainings followed the same protocol. Only second antibody didn’t show any positive stains (Left panel). After adding the primary antibody, Ki 67 was positively stained in the nucleus indicating proliferating cells (Right panel). Scale bar indicates 100 μm.

**Supplementary Figure S13. Specificity of in-situ apoptosis staining (TUNEL).** All the stainings followed the same protocol. dH2O, used as negative control, didn’t show any positive stains (Left panel). After adding TdT Enzyme incubation, TUNEL was positively stained in the nucleus indicating apoptotic cells (Right panel). Scale bar indicates 100 μm.

***Supplementary Method: Molecular dynamics and binding free-energy estimates***

PDB files of complexes of DOX and HA were prepared as complexes with *packmol (1)* with HA as a (001) crystal slab. DOX was moved to the center of the HA crystal slab for centering the position over the HA slab using VMD (2). The PDB files of HA as well as force field parameters were obtained from the Amber Interface Force Field (IFF) [*INTERFACE MD. https://bionanostructures.com/interface-md/(2016)*]. The atomic charges of HA were obtained from MOL2 files generated with the car_to_files.py of *Ambertools20.* Considerations of the pH range 7.4-5.5 were taken into account for both DOX and HA. The pK_a_ is 7.34 for the phenol moiety of DOX and 8.46 for the amine. The amine group is protonated below a pH=8.46. The acid dissociation constants (pK_a_) of phosphoric acid are 2.14, 7.20, and 12.37. The phosphate is protonated as H_3_PO_4_ below pH=2.4, and as H_2_PO_4_^-^above at lysozyme pH 5.5. At physiological pH 7.4, the hydroxyapatite crystal has a mixture of phosphoric acid with HPO_4_^-2^ and H_2_PO_4_^-^ so the H_2_PO_4_^-^ was used to model the hydroxylated surface at this pH. The phosphate becomes deprotonated at pH ~12.37 where the solid lattice is Ca_10_(PO_4_)_8_(OH)_2_ (3). In order to keep the stoichiometric chemistry, half of the calcium must be removed from the surface when the phosphate is protonated. AM1-BCC charges for DOX were derived from the *antechamber* program. The *gaff2* keyword was used to derive atomic charges with net zero charge. The *parmchk2* program was used to assign missing force field parameters for the GAFF force field for DOX using the *antechamber* module from *AmberTools20 (4)*.

All free-energy simulations (FES) were performed with *NAMD 2.14* (5) in the NPT ensemble with periodic boundary conditions using Particle Mesh Ewald summation for 10 ps per $\lambda$ with a total sampling of 50 ps. The sampling time was chosen with respect to the systems’ rather few degrees of freedom. All structures were minimized by 1000 steps of minimization with a restraint of 1000 kcal mol^-1^Å^-2^ on all atoms. A first equilibration was performed in the NVT ensemble for 1 ps with DOX unrestrained. A second equilibration was performed in the NPT ensemble for 1 ps with both DOX and water molecules unrestrained whereas HA was still restrained with 1000 kcal mol^-1^ Å^-2^ as a crystal slab. The MD simulations were performed using cut-off electrostatics and Lennard–Jones interactions with a cut-off radius of 12 Å, respectively. The pair list distance for the long-range electrostatics was 14 Å.

Amber topology files and input coordinate files of HA were prepared using the *tleap* module of *AmberTools20.* The non-standard force field parameters for hydroxyapatite were obtained from the Amber Interface Force Field (IFF). The Lennard–Jones 12-6 van der Waals (vdW) radii were updated from parameters published by Tzu-Jen Lin (3). This included parameters for the phosphoric acids in hydroxyapatite and the calcium ion. The Amber TIP3P force field was used for the water molecules by solvation using the *tleap* module.  A number of 10618 and 10829 water molecules were added to the simulation box surrounding the DOX–HA complexes at a distance of 20 Å from the complex for the two different pH levels, respectively.

The *amber* keyword was used to read Amber topology and coordinate files in NAMD, i.e. the output from *tleap*. In the MD simulations, bonds involving hydrogen atoms were constrained with the *rigidBonds* keyword (corresponds to *ntc=2, ntf=2* in AMBER). The configuration file was adapted to resemble the Amber software defaults in NAMD, and the configuration files are provided below. In particular this adaption includes full electrostatics and non-bonded interaction evaluation at each timestep. As Amber topology was used an adaptation to resemble Amber software defaults were made that includes rescaling 1-4 interactions by a factor of 1/1.2 and constraining hydrogen bonds with the SHAKE algorithm i.e. using the RATTLE algorithm for water molecules instead. Moreover, this adaptation involves applying an analytical tail correction to the vdW energy and virial that is equal to the amount lost due to switching and cutoff of the Lennard–Jones potential, removing the center of mass drift due to Particle Mesh Ewald (PME) and employing the Langevin thermostat. In all simulations, the temperature was kept constant at 300 K using a Langevin integrator with a collision frequency of 2 ps. A Langevin barostat with the pressure kept constant at 1 atm (1.01325 bar) was used in the NPT ensemble having a relaxation time of 100 ps and a compressibility of 4.57e-5. The occupancy column values of a PDB file from building the prmtop topology file, was used to configure the restraints implemented with the *conskfile* keyword. It should be noted that the size of the periodic cell was the same for each alchemical transformation but different in the bound state and the free state as was required by the NAMD software. The periodic cell basis vectors and cell origin were determined specifically for each simulation for use in the NAMD configuration files.

Absolute binding free energies were calculated for DOX by turning off both the electrostatics and the Lennard–Jones interactions when transforming DOX in the coupling parameter $\lambda$. FES calculations were run with the single topology scheme as implemented in NAMD. The binding free-energy of DOX to HA can be calculated from two sets of alchemical transformations i.e. decoupling the non-bonded interactions of DOX bound to HA and decoupling the non-bonded interactions of DOX in the water box according to a thermodynamic cycle connecting the bound and the free states, see figure below. From these two alchemical transformations the binding free-energy, ΔG_bind_, can be calculated according to Eq. 1:

$\Delta G_{bind} =\Delta G_{bind}(L_{0}) - \Delta G_{bind}(L_{1}) = \Delta G_{b} - \Delta G_{f}$   (1)

**
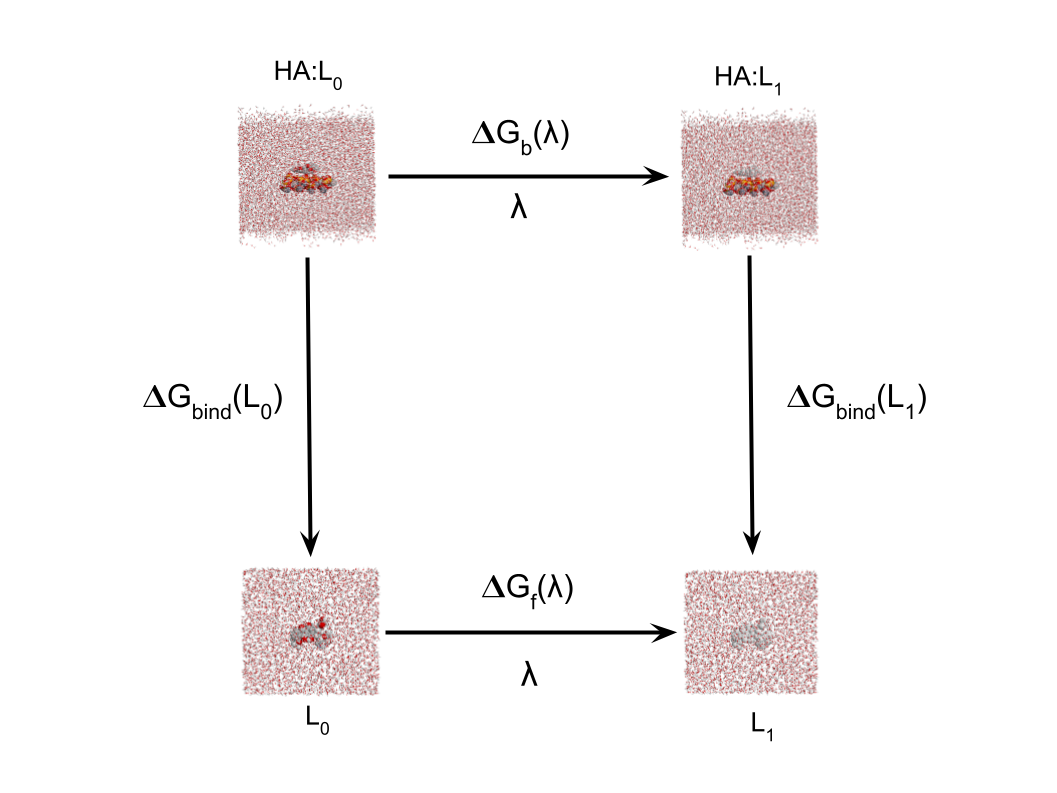
**

where subscript *b* and *f* denote bound and free states, respectively. FES calculations in the forward direction of the alchemical transformation were performed for a number of 5 $\lambda$-values with an interval of 0.2 in the coupling parameter $\lambda$, 0.1, 0.3, 0.5, 0.7, 0.9, using a linear transformation of the potentials according to Eq. 2.

$V = \left( 1-\lambda\right)V_{0} + \lambda V_{1}$  (2)

The *parseFEP* plugin (6) was used to calculate the free-energies of ΔG_b_  and ΔG_f_ , respectively. The absolute free-energies were estimated using bidirectional sampling with multi-state Bennett-acceptance ratio (MBAR) (7) i.e. turning off the non-bonded interactions in the forward direction $\lambda=0\to1$ and turning on the non-bonded interactions of DOX in the backwards direction $\lambda=1 \to0$.

**References:**

1. Martínez L, Andrade R, Birgin EG, Martínez JM. PACKMOL: a package for building initial configurations for molecular dynamics simulations. J Comput Chem. 2009;30(13):2157-64.

2. Humphrey W, Dalke A, Schulten K. VMD: visual molecular dynamics. J Mol Graph. 1996;14(1):33-8, 27-8.

3. Lin T-J, editor Force field parameters and atomistic surface models for hydroxyapatite and analysis of biomolecular adsorption at aqueous interfaces2013.

4. D.A. Case HMA, K. Belfon, I.Y. Ben-Shalom, S.R. Brozell, D.S. Cerutti, T.E. Cheatham, III, V.W.D. Cruzeiro, T.A. Darden, R.E. Duke, G. Giambasu, M.K. Gilson, H. Gohlke, A.W. Goetz, R. Harris, S. Izadi, S.A. Izmailov, C. Jin, K. Kasavajhala, M.C. Kaymak, E. King, A. Kovalenko, T. Kurtzman, T.S. Lee, S. LeGrand, P. Li, C. Lin, J. Liu, T. Luchko, R. Luo, M. Machado, V. Man, M. Manathunga, K.M. Merz, Y. Miao, O. Mikhailovskii, G. Monard, H. Nguyen, K.A. O’Hearn, A. Onufriev, F. Pan, S. Pantano, R. Qi, A. Rahnamoun, D.R. Roe, A. Roitberg, C. Sagui, S. Schott-Verdugo, J. Shen, C.L. Simmerling, N.R. Skrynnikov, J. Smith, J. Swails, R.C. Walker, J. Wang, H. Wei, R.M. Wolf, X. Wu, Y. Xue, D.M. York, S. Zhao, and P.A. Kollman. Amber 2020. University of California, San Francisco. 2020.

5. Phillips JC, Hardy DJ, Maia JDC, Stone JE, Ribeiro JV, Bernardi RC, et al. Scalable molecular dynamics on CPU and GPU architectures with NAMD. The Journal of Chemical Physics. 2020;153(4):044130.

6. Liu P, Dehez F, Cai W, Chipot C. A Toolkit for the Analysis of Free-Energy Perturbation Calculations. J Chem Theory Comput. 2012;8(8):2606-16.

7. Shirts MR, Chodera JD. Statistically optimal analysis of samples from multiple equilibrium states. The Journal of Chemical Physics. 2008;129(12):124105.
